# Imidazolium salts carrying two positive charges: design, synthesis, characterization, molecular docking, antibacterial and enzyme inhibitory activities

**DOI:** 10.3389/fcimb.2025.1579916

**Published:** 2025-07-18

**Authors:** Ilter Demirhan, Adem Necip, Erkan Oner, Nalin Gumuscu, Ozlem Demirci, Yetkin Gok, Nebiye Yentur Doni, Mesut Işık, Mithun Rudrapal, Johra Khan, Randa Mohammad Ibrahim

**Affiliations:** ^1^ Department of Electronic-Otomation, Biomedical Device Technology Program, Vocational School of Health Services, Harran University, Sanlıurfa, Türkiye; ^2^ Department of Pharmacy Services, Vocational School of Health Services, Harran University, Sanlıurfa, Türkiye; ^3^ Department of Biochemistry, Faculty of Pharmacy, Adıyaman University, Adıyaman, Türkiye; ^4^ Department of Dental Services, Vocational School of Health Services, Harran University, Sanlıurfa, Türkiye; ^5^ Department of Medical Biology, Cerrahpasa Medical Faculty, Istanbul University-Cerrahpasa, Istanbul, Türkiye; ^6^ Department of Chemistry, Faculty of Arts and Science, Inönü University, Malatya, Türkiye; ^7^ Department of Medical Microbiology, Faculty of Medicine, Harran University, Salnlıurfa, Türkiye; ^8^ Department of Bioengineering, Faculty of Engineering, Bilecik Şeyh Edebali University, Bilecik, Türkiye; ^9^ Department of Pharmaceutical Sciences, School of Biotechnology and Pharmaceutical Sciences, Vignan’s Foundation for Science, Technology and Research, Guntur, India; ^10^ Department of Medical Laboratory Sciences, College of Applied Medical Laboratory Sciences, Majmaah University, Al Majma’ah, Saudi Arabia; ^11^ Health and Basic Science Research Center, Majmaah University, Al Majma’ah, Saudi Arabia; ^12^ Department of Microbiology and Immunology, Veterinary Sciences Institute, National Research Center, Giza, Egypt

**Keywords:** N-heterocyclic carbene, imidazolium salt, acetylcholinesterase, antimicrobial, anti-Alzheimer

## Abstract

**Introduction:**

The discovery of alternative drugs has gained importance due to the many side effects of these drugs used for treatment.

**Methods:**

Herein, the synthesis of a series of unsymmetrical imidazolium salts containing 4-acetylphenyl/4-formylphenyl and bioactive heterocyclic groups such as morpholine, piperidine, pyrrole or pyridine was reported. 4-(1-*H*-imidazol-1-yl)acetophenone and 4-(1-*H*-imidazol-1-yl)benzaldehyde were used as salt precursors. Alkyl halides containing heterocyclic groups such as 2-morpholinoethyl hydrochloride, 2-pyrrolidinoethyl hydrochloride, 2-piperidinoethyl hydrochloride and pyridin-2-ylmethyl bromide hydrobromide were used. Thus, there are two positively charged nitrogens in the structure of these salts synthesized by the quaternization method. The structures of all salts were fully characterized by ^1^H, ^13^C NMR, FTIR spectroscopic and elemental analysis methods. the a series of imidazolium salts (1a-d and 2a-d) were designed, synthesized and fully characterized by spectroscopic methods.

**Results:**

The inhibitory effect against AChE of the series compounds was evaluated as *in vitro* and in silico studies. The results indicated that the compounds showed remarkably potent inhibitory effects on AChE with *K*_I_ values ranging from 0.63 ± 0.04 μM to 11.23 ± 1.05 μM and IC_50_ values spanning from 0.82 ± 0.06 μM to 14.75 ± 0.82 μM. The antimicrobial activities of the synthesized compounds were measured by inhibition of bacterial growth expressed as minimum inhibitory concentration (MIC) values. It was observed that the synthesized compounds exhibited antimicrobial activity especially against Gram negative bacteria. In addition, the results of molecular docking studies of bacteria supported our antimicrobial results.

**Conclusions:**

The results suggested that the synthesized compounds showed the potential to be antimicrobial and acetylcholinesterase inhibitors.

## 1 Introduction

Heterocyclic compounds are organic compounds that contain at least one hetero atom (such as nitrogen, sulfur, oxygen) and are found in the structure of drugs used in the treatment of many diseases due to their biological properties. Such compounds are found as subunits in many natural compounds such as vitamins, hormones, alkaloids, dyes and a wide variety of biomolecules including various other chemicals (Satheesh et al., 2020). A large number of natural and synthetic heterocyclic compounds are being investigated due to their pharmacologically active molecules. *N*-heterocyclic compounds containing at least one nitrogen atom in the ring system exhibit a wide range of biological activities such as antibacterial, antiviral, antifungal, antituberculosis, anti-inflammatory, antidepressants, antimycobacterial, antipyretics, analgesics, anticonvulsants, antihistamines, antiplasmodial, antidiabetic, anticancer. According to FDA database, approximately 60% of small molecule drugs are composed of nitrogen-based heterocyclic compounds, such as Metronidazole, Sovaldi, Abilify, Diazepam, Nexium, Crestor, Chlorpromazine, Isoniazid, Captopril, Chloroquine, and Clotrimazole (Aktas et al., 2018; Tessier et al., 2018; Taslimi et al., 2020). This shows the structural importance of *N*-heterocyclic compounds in drug design and drug discovery. *N*-heterocyclic compounds in biologically active compounds can maintain their stability and operational efficiency in the human body for a longer time when taken as drugs. Their biological activity is based on the ability of the ring nitrogen atom to easily bind to DNA by hydrogen bonding interaction.

Imidazole and benzimidazole derivatives have several favorable properties, such as excellent bioavailability, good tissue penetration and permeability, and relatively low incidence of side and toxic effects. Thus, these compounds demonstrate significant development potential in organic chemistry, coordination chemistry, materials science, and medicinal chemistry (Ardugenyo et al., 1999; Wisniak, 2009). Imidazolium salts are structures in which alkyl groups are attached to the two nitrogen atoms in the imidazole ring. The imidazole ring is ubiquitous in nature and plays a critical role in many structures and functions in the human body (Wanzlick and Schönherr, 1968). The functionality of the imidazole ring is a ligand with strong σ-electron donor and weak π-acceptor properties, forming organometallic compounds by bonding with almost all metals. In addition to the very important catalytic properties of these complexes, they are important in medicinal chemistry due to their ability to interact with drugs and proteins (such as hydrogen bonding, electrostatic interactions) (Garrison and Youngs, 2005; Van Veldhuizen et al., 2005; Garber et al., 2000; Ofele, 1968). The most important and attractive features of imidazolium salts are the flexibility in the design of their physical, chemical and biological properties by independently changing the structural diversity in the ring, the properties of the cation and anion. For this reason, these compounds have become good templates for various applications in chemistry and related industries, cosmetics, nanotechnology and pharmaceuticals (Amyes et al., 2004; Van Veldhuizen et al., 2003; Arduengo et al., 1999). Many research groups have recently investigated benzimidazole, imidazole and imidazoline salts as enzyme inhibitors, which exhibit inhibitory activities against various metabolic enzymes and potent cytotoxic activity against cancer cells (Wang et al., 2005; Glorius et al., 2002; Yang et al., 2001; Gardiner et al., 1999; Herrmann et al., 1996; Hussey, 1983).

**Figure 1:**
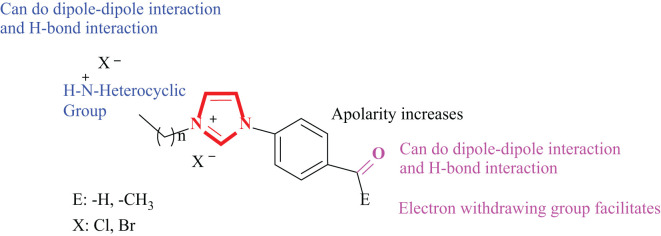
Structural features of imidazolium salts carrying two positive charges.

The importance of these compounds in drug design as active drug substances stems from their ability to change their biological and physicochemical properties, pharmacokinetics and toxicological profiles (Jamil, 2019; Marion et al., 2009). In recent years, a pharmacophore hybrid approach for the discovery of new and highly bioactive pharmaceutical ingredients has become an effective and widely used trend in the field of drug discovery. Recently, when the literature is examined, it is seen that there are fewer studies on the enzyme inhibition activities of imidazolium salts (Chauvin, 1995). Our workgroup has recently studied the inhibitory activities of various alkyl/aryl group-containing benzimidazolium salts on some metabolic enzymes (Pernak et al., 2004; Haziz et al., 2019; Iqbal et al., 2013). In this study, it was thought that imidazolium salts carrying two positive charges could be suitable alternatives to quaternary ammonium compounds, which are widely used as antiseptics, disinfectants, antimicrobials and antimicrobials, with adjusted hydrophobic, hydrophilic and electrostatic properties (Selvarajoo et al., 2017). Thus, in our study, it includes the synthesis and biological properties of two series of imidazolium salts containing bioactive piperidine, morpholine, pyrrole, pyridine heterocyclic rings by alkylation of the third nitrogen atoms in the 1-(4-acethyl/formylbenzyl) imidazole ring (**Figure 1**). The structures of the imidazolium salts carrying two positive charges were characterized using appropriate spectroscopic methods (^1^H NMR, ^13^C NMR and FTIR) and elemental analysis techniques. The inhibitory activities of the synthesized imidazolium salts against acetylcholine esterase enzyme were investigated. Additionally, the antimicrobial properties of the synthesized salts were also investigated.

The cholinergic hypothesis, one of the leading theories in Alzheimer’s disease (AD) pathogenesis, suggests that the inhibition of cholinesterase enzymes, particularly acetylcholinesterase (AChE), is a key therapeutic strategy to counteract the decline in acetylcholine levels in the brain. The AChE, a membrane-bound enzyme present in various tissues, including the brain, plays a critical role in hydrolyzing acetylcholine, a neurotransmitter essential for cognitive function (Thacker, 2003; Işık, 2019; Durgun et al., 2020). The AChE is a highly selective enzyme for acetylcholine, ensuring rapid and precise termination of synaptic transmission, and is therefore considered the principal target in the development of therapeutic agents for neurodegenerative disorders such as AD (Dastan et al., 2017; Lolak et al., 2020; Mukhametgalieva et al., 2022). Current AChE inhibitors, such as galantamine, rivastigmine, donepezil, and tacrine, are widely used in AD treatment. However, these drugs are associated with limitations, including short half-lives and adverse side effects such as nausea, vomiting, and gastrointestinal disturbances, while providing only symptomatic relief or slowing disease progression (Lolak et al., 2022; Prasasty et al., 2018; Jia et al., 2013; Fernández-Bachiller et al., 2010). Consequently, the design and discovery of novel AChE inhibitors with improved efficacy and reduced side effects remain crucial for advancing AD therapeutics.

The blood-brain barrier protects the central nervous system (CNS) with its structure consisting of microvascular endothelial cells, pericytes and astrocytes; this structure maintains homeostasis by selectively controlling molecules entering and leaving the brain (Gloor et al., 2001). Outside the CNS, interactions between nerve endings and immune cells are markedly increased in inflammatory processes, especially in areas exposed to environmental factors such as mucosal surfaces. The detection of bacterial structures in the CNS tissues of Alzheimer’s patients raises the question of the routes by which these microorganisms reach neuronal regions. In this context, the presence of intestinal microbiota-derived proinflammatory neurotoxins (e.g. lipopolysaccharide - LPS) that can cross the gastrointestinal mucosa and enter the circulation suggests that cellular adhesion structures and mechanisms may play an important role in the transport of these harmful molecules (Kraneveld et al., 2014; Panza et al., 2019).

In this study, a series of imidazolium salts (1a-d and 2a-d) were designed, synthesized and fully characterized by spectroscopic methods. The inhibitory activity of the series against AChE was evaluated by *in vitro* and *in silico* studies.

## 2 Materials and methods

### 2.1 Experimental

Imidazolium salts containing carbonyl group and a second heterocyclic group outside the main imidazole ring were synthesized in argon atmosphere by using the standard Schlenk tube technique. Acetonitrile used in salt synthesis was purified from oxygen by bubbling argon for 10 minutes, and the water it contained was removed on activated 4Å molecular sieves. The chemical used in this study was purchased from Abcr, Acros, Isolab and Sigma-Aldrich Chemical Co. It was purchased by and used as received without any purification process. The melting points of the synthesized salts were determined using the Electrothermal-9200 device. FTIR spectra were recorded in the range of 400–4000 cm^−1^ on a Perkin Elmer Spectrum 100 FTIR spectrometer. ^1^H and ^13^C NMR spectra were recorded using a Bruker Avance III 400 MHz NMR spectrometer (400 MHz for ^1^H and 100 MHz for ^13^C NMR) using DMSO-d6 solvent.

### 2.2 Synthesis

*1-(4-acetylphenyl)-3-(2-morpholino)ethylimidazolium chloride hydrochloride, 1a*

1-(4-acetylphenyl)imidazole (1 mmol) and 2-morpholinoethyl chloride hydrochloride (1 mmol) were heated in acetonitrile (4 mL) for 24 h and at 80°C. A white solid precipitated and the solvent was then removed in vacuo. This solid was washed with diethyl ether (2 x 10 mL). The crude product was recrystallized from ethyl alcohol/diethyl ether (1:3) at room temperature to give white imidazolium salt. Yield: 75%. ^1^H NMR (400 MHz, CDCl_3_, 298 K), δ: 2.58 (s, 3H, -NC_6_H_4_COCH_3_); 3.21 and 3.95 (t, 4H, *J*: 6.3 and 6.4 Hz, NCH_2_CH_2_N); 3.07 (m, 4H, morpholino -CH_2_); 3.99 (t, 4H, *J*: 4.4 Hz, morpholino-CH_2_); 7.25 and 7.33 (s, 2H, imidazol-4,5-CH); 7.49 and 8.04 (d, 4H, *J*: 8.5 Hz, -NC_6_H_4_COCH_3_); 8.20 (s, 1H, NCHN). ^13^C NMR (100 MHz, CDCl_3_, 298 K), δ: 26.2 (-C_6_H_4_COCH_3_); 37.3 and 59.0 (NCH_2_CH_2_N); 52.8 and 64.3 (morpholino-CH_2_); 121.0, 130.4, 136.2 and 140.4 (Ar-C); 142.7 (NCHN); 196.4 (-NC_6_H_4_COCH_3_).

*1-(4-acetylphenyl)-3-(2-piperidino)ethylimidazolium chloride hydrochloride, 1b*

Yield: % 72. M.p.: 158-159°C; ν(C=O): 1672 cm^−1^; ν(CN): 1605 cm^−1^. ^1^H NMR (400 MHz, CDCl_3_, 298 K), δ: 2.59 (s, 3H, -NC_6_H_4_COCH_3_); 2.85 and 3.81 (s, 8H, piperidino-CH_2_); 3.40 and 4.00 (t, 4H, *J*: 6.5 Hz, NCH_2_CH_2_N); 7.35 and 7.40 (s, 2H, imidazol-4,5-CH); 7.54 and 8.06 (d, 4H, *J*: 8.3 and 8.2 Hz, NC_6_H_4_COCH_3_); 8.81 (s, 1H, NCHN); 12.90 (s, 1H, HCl). ^13^C NMR (100 MHz, CDCl_3_, 298 K), δ: 23.2 and 54.3 (piperidino-CH_2_); 26.7 (-C_6_H_4_COCH_3_); 37.8 and 56.1 (NCH_2_CH_2_N); 121.4, 127.2, 130.5, 136.9 and 137.6 (Ar-C); 142.4 (NCHN); 196.4 (-NC_6_H_4_COCH_3_).

*1-(4-acetylphenyl)-3-(2-pyrrolidino)ethylimidazolium chloride hydrochloride, 1c*

Yield: % 67. M.p.: 233-234°C; ν(C=O): 1682 cm^−1^; ν(CN): 1557 cm^−1^. ^1^H NMR (400 MHz, CDCl_3_, 298 K), δ: 2.58 (s, 3H, -NC_6_H_4_COCH_3_); 3.28 and 4.04 (t, 4H, *J*: 6.5 Hz, NCH_2_CH_2_N); 1.84, 2.20, 2.71 and 3.56 (s, 10H, pyrrolidino-CH_2_); 7.29 and 7.36 (s, 2H, imidazol-4,5-CH); 7.54 and 8.06 (d, 4H, *J*: 8.6 Hz, NC_6_H_4_COCH_3_); 8.49 (s, 1H, NCHN); 12.62 (s, 1H, HCl). ^13^C NMR (100 MHz, CDCl_3_, 298 K), δ: 21.9, 23.0 and 53.9 (pyrrolidino-CH_2_); 26.6 (-C_6_H_4_COCH_3_); 36.6 ve 58.2 (NCH_2_CH_2_N); 118.3, 121.2, 128.3, 130.5 and 136.5 (Ar-C); 139.9 (NCHN); 196.4 (-NC_6_H_4_COCH_3_).

*1-(4-acetylphenyl)-3-(pyridin-2-yl)methylimidazolium bromide hydrobromide, 1d*

1-(4-acetylphenyl)-3-(pyridin-2-yl)methylimidazolium bromide hydrobromide (1d) synthesized in our work carries an additional charge compared to the previously reported salt, 3-(4-acetylphenyl)-1-(2-pyridylmethyl)imidazolium bromide (Ibrahim and Bala, 2016). Yield: % 64. M.p.: 298-299°C; ν(C=O): 1636 cm^−1^; ν(CN): 1501 cm^−1^. ^1^H NMR (400 MHz, CDCl_3_, 298 K), δ: 2.67 (s, 3H, -NC_6_H_4_COCH_3_); 5.77 (s, 2H, NCH_2_C_5_H_4_N-2); 7.70 (d, 1H, *J*: 7.7 Hz, imidazol-4,5-CH); 8.52 (s, 1H, imidazol-4,5-CH); 8.02 and 8.23 (d, 4H, *J*: 8.8 Hz, NC_6_H_4_COCH_3_); 10.26 (s, 1H, NCHN). ^13^C NMR (100 MHz, CDCl_3_, 298 K), δ: 27.5 (-C_6_H_4_COCH_3_); 54.0 (s, 2H, NCH_2_C_5_H_4_N-2); 121.6, 122.3, 123.8, 124.7, 124.8, 130.6, 137.2, 137.8, 138.3, 149.1 and 152.9 (Ar-C); 139.3 (NCHN); 197.5 (-NC_6_H_4_COCH_3_).

*1-(4-formylphenyl)-3-(2-morpholino)ethylimidazolium chloride hydrochloride, 2a*

Yield: % 78. ^1^H NMR (400 MHz, CDCl_3_, 298 K), δ: 3.42 and 4.04 (t, 4H, *J*: 6.9 and 6.8 Hz, NCH_2_CH_2_N); 3.21 (s, 2H, morpholino-CH_2_); 3.84-3.88 (m, 6H, morpholino-CH_2_); 7.37 and 8.06 (s, 2H, imidazol-4,5-CH); 7.99 and 8.09 (d, 4H, *J*: 8.6 Hz, -NC_6_H_4_CHO); 10.02 (s, 1H, NCHN); 10.11 (s, 1H, -NC_6_H_4_CHO); 10.28 (s, 1H, NCHN). ^13^C NMR (100 MHz, CDCl_3_, 298 K), δ: 37.8 and 56.9 (NCH_2_CH_2_N); 31.2, 36.3, 51.8 and 63.8 (morpholino-CH_2_); 119.0, 121.2, 122.9, 131.7, 135.2 and 162.8 (Ar-C); 141.9 (NCHN); 192.6 (-NC_6_H_4_CHO).

*1-(4-formylphenyl)-3-(2-piperidino)ethylimidazolium chloride hydrochloride, 2b*

Yield: % 71. M.p.: 239-240°C; ν(C=O): 1700 cm^−1^; ν(CN): 1604 cm^−1^. ^1^H NMR (400 MHz, CDCl_3_, 298 K), δ: 4.40 and 4.79 (s, 3H, NCH_2_CH_2_N); 4.06 (t, 1H, *J*: 7.0 Hz, NCH_2_CH_2_N); 1.79, 2.97 and 3.66 (s, 10H, piperidino-CH_2_); 7.96 (d, 1H, *J*: 8.2 Hz, imidazol-4,5-CH); 7.36 (s, 2H, imidazol-4,5-CH); 7.54 and 8.06 (d, 4H, *J*: 8.6 Hz, NC_6_H_4_CHO); 8.47 (s, 1H, imidazol-4,5-CH); 10.13 (s, 1H, -NC_6_H_4_CHO); 10.32 (s, 1H, NCHN); 11.48 (s, 1H, HCl). ^13^C NMR (100 MHz, CDCl_3_, 298 K), δ: 19.0, 21.8, 22.7, 52.7 and 52.9 (piperidino-CH_2_); 40.9 and 56.5 (NCH_2_CH_2_N); 118.3, 120.9, 121.4, 122.9, 124.1, 131.7 and 136.9 (Ar-C); 139.4 (NCHN); 192.7 (-NC_6_H_4_CHO).

*1-(4-formylphenyl)-3-(2-pyrrolidino)ethylimidazolium chloride hydrochloride, 2c*

Yield: % 78. ^1^H NMR (400 MHz, CDCl_3_, 298 K), δ: 1.92, 2.01, 3.12 and 3.65 (s, 8H, pyrrolidino-CH_2_); 3.68 and 4.76 (s, 4H, NCH_2_CH_2_N); 8.19 and 8.47 (s, 2H, imidazol-4,5-CH); 8.09 and 8.20 (d, 4H, *J*: 8.5 and 8.7 Hz, NC_6_H_4_CHO); 10.12 (s, 1H, -NC_6_H_4_CHO); 10.41 (s, 1H, NCHN); 11.65 (s, 1H, HCl). ^13^C NMR (100 MHz, CDCl_3_, 298 K), δ: 19.0, 23.3, 46.0 and 53.6 (pyrrolidino-CH_2_); 40.9 and 56.5 (NCH_2_CH_2_N); 121.7, 123.1, 124.0, 137.0 and 137.9 (Ar-C); 139.4 (NCHN); 192.7 (-NC_6_H_4_CHO).

*1-(4-formylphenyl)-3-(pyridin-2-yl)methylimidazolium bromide hydrobromide, 2d*

Yield: % 73. M.p.: 239-240°C; ν(C=O): 1696 cm^−1^; ν(CN): 1602 cm^−1^. ^1^H NMR (400 MHz, CDCl_3_, 298 K), δ: 6.13 (s, 2H, NCH_2_C_5_H_4_N-2); 7.99 and 8.44 (s, 2H, imidazol-4,5-CH); 8.10 and 8.19 (d, 4H, *J*: 8.3 Hz, NC_6_H_4_CHO); 10.10 (s, 1H, NCHN); 10.13 (s, 1H, NC_6_H_4_CHO); 11.24 (s, 1H, HBr). ^13^C NMR (100 MHz, CDCl_3_, 298 K), δ: 56.5 (NCH_2_C_5_H_4_N-2); 121.1, 121.9, 123.0, 127.6, 131.1, 131.6, 135.7 and 136.8 (Ar-C); 139.5 (NCHN); 192.7 (-NC_6_H_4_CHO).

### 2.3 Biochemical studies

#### 2.3.1 Measurement of AChE activity

AChE activity was determined by spectroscopic method using acetylthiocholine iodide as substrate (Ellman et al., 1961; Işık and Beydemir, 2021). Reaction mixtures contained the 50 μL of 5,5′-dithio-bis(2-nitro-benzoic)acid compound (DTNB) and 100 μL of Tris–HCl solution (1 M, pH 8.0), and 20 μL AChE solution in total volume of 1 mL. The mixture was then incubated and mixed for 15 min at 30°C. Next, The reaction was started by adding 50 μL of acetylthiocholine iodide, and carried out for 5 min at 30°C. After adding substrate, the change in absorbance at 0 and 5 minutes was recorded at 412 nm.

#### 2.3.2 *In vitro* inhibition studies

AChE activities were determined at 30°C by using five concentrations of acetylthiocholine iodide and three concentrations of compounds. The inhibition percentage for each compound, the IC_50_ values, the Ki values with Lineweaver-Burk curves and the inhibition types were determined to reveal the mechanism of inhibition (Demir et al., 2017; Türkeş et al., 2021).

### 2.4 Antimicrobial test

This study used standard strains of bacteria known to be resistant to antibiotics. These isolates were identified as three gram-positive bacteria: *Bacillus subtilis, Staphylococcus aureus* (ATCC 29213) and *Enterococcus faecalis* (ATCC 29212); and two Gram-negative bacteria: *Escherichia coli* (ATCC 25922) and *Pseudomonas aeruginosa* (ATCC 27853). The strains were grown in blood medium one night before the broth microdilution method was used. They were then passaged and stored in tryptic soy broth (TSB) at -20°C for repeat experiments. Bacterial strains were passaged in blood medium and incubated in an oven at 35 ± 2°C for 18–24 hours. At least 3–5 similar colonies were selected from the culture plate. These were picked up with a loop and transferred to 4–5 mL of liquid medium (e.g. tryptic soy broth). The broth was incubated at 35°C until the turbidity reached McFarland 0.5 (approximately 2–6 hours). The density of the culture in the broth was determined by preparing a bacterial solution with a McFarland density of 0.5. The microdilution method was used to determine MIC values. Standards were dissolved in DMSO. Positive controls were prepared with penicillin, fluconazole and gentamicin.

It was prepared at concentrations of 1, 2, 4, 8, 16, 32, 64, 128, 256 and 512 µg/mL. 1% DMSO was used as a solvent control. Compounds were spiked at initial test control concentrations of 250 µg/mL in MHB. MHB was added to all wells except the first well. 100 µl of compounds were added to the first well. Serial dilutions were then made and diluted to the minimum concentration (1 µg/ml). Then 0.5 McFarland was prepared from bacteria incubated in MHB for 1 night. Then 50 µl of bacterial suspension was added to each well. The plates were incubated at 37°C for 24 hours. The microplates were then read in the spectrophotometer at a wavelength of 620 nm.

### 2.5 Minimum inhibition on konsantrasyon

100 µl of MHB was added to each well of a 12-well U-bottom microplate. 100 μL of the previously prepared NHC precursor drug was taken and transferred to the first well, and 100 μL was taken again by pipetting and transferred to the second well. In this way, dilutions were made sequentially up to the 11^th^ well. No NHC precursor was added to the 12th well, which was used as a positive control. Then 100 μL of the prepared 5 x 105 CFU/mL bacterial suspension was taken and added to all wells without adding to the 11th well. The 11th well was used as a negative control (Demirhan et al., 2024). The same study was performed for all NHC precursors. The microplates were then incubated at 35°C for 24 hours. The lowest concentration at which no growth occurred for all strains was determined as the MIC.

### 2.6 *In silico* details

***S*** *. aureus* (PDB ID: 1MVT), *E. coli* (PDB ID: 4XO8), *E. faecalis* PDB ID: 6ORI and *P. aeruginosa* PDB ID: 4JVI protein structures and molecular docking studies were performed on the synthesized compounds using Discovery Studio (version 2021) (Biovia, 2021). The structures of compounds 1a-2d were drawn using ChemBioDraw Ultra [Chemical Structure Drawing Standard; Cambridge Soft Corporation, USA (2010)] and then energetically minimized using Discovery Studio. The co- crystallized protein-ligand complex structure (pdb IDs 1MVT, 4XO8, 6ORI and 4JVI) was downloaded from the Protein Data Bank and prepared according to the requirements of the docking study, such as addition of hydrogen atoms and removal of water/impurities. The binding site was defined according to the volume occupied by the bound ligand in the ‘Define and edit binding site’ tools of DS 2019. Other parameters were left at their default values. Compounds 1a-2d were docked to the receptor using the Autodock 4.2.6 and Autodock Vina packages (Trott and Olson, 2009; Pettersen et al., 2004; Oner et al., 2023).

## 3 Results

### 3.1 Characterization results of synthesized compounds

**Figure 2:**
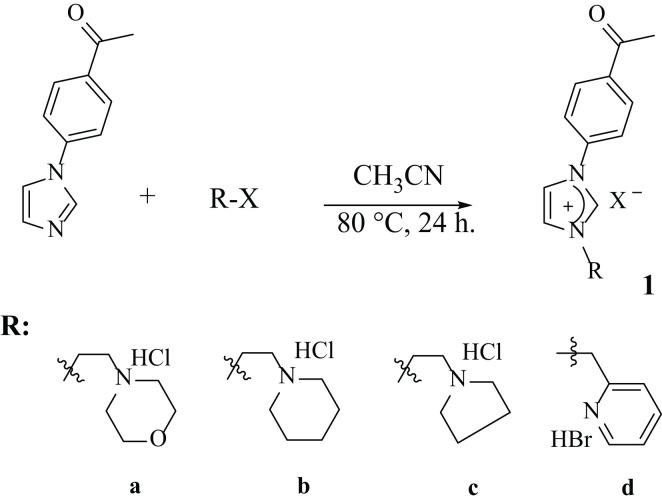
Synthesis of imidazolium salts containing 4-acetylphenyl.

**Figure 3:**
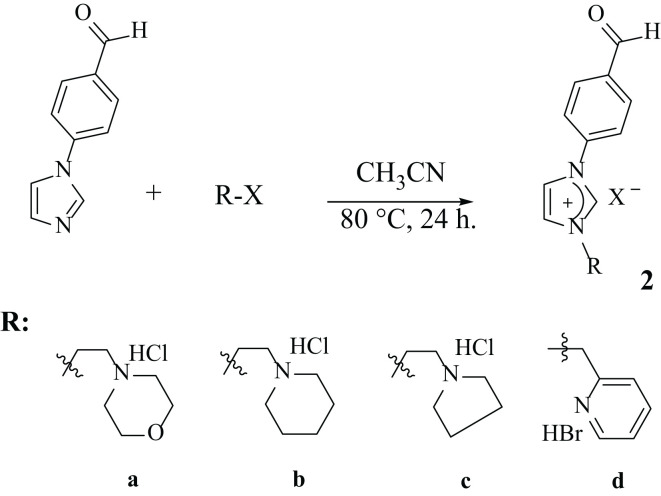
Synthesis of imidazolium salts containing 4-formylphenyl.

**Figure 4:**
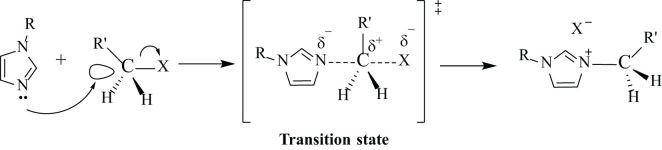
Synthesis mechanism of imidazolium salts (1a-d and 2a-d).

Unsymmetrical new imidazolium salts (1a-d and 2a-d) containing 4-acetylphenyl or 4-formylphenyl groups were prepared according to the literature method by the reactions of 4-(1-*H*-imidazol-1-yl)acetophenone/4-(1-*H*-imidazol-1-yl)benzaldehyde with various heterocyclic ring alkyl halides in acetonitrile for 24 h at 80 °C (**Figures 2**, **3**). Double positively charged imidazolium salts were synthesized via a quaternization reaction proceeding through an S_N_2 mechanism (**Figure 4**). In this S_N_2 mechanism, *N*-alkylimidazole acts as the nucleophile. Both the *N*-alkylimidazole and the alkyl halide significantly influence the reaction rate (Messali, 2014).

### 3.2 Enzyme inhibition (AChE) results

The synthesized derivatives (1a-d and 2a-d) with a series of imidazolium salts demonstrated significant inhibitory activity against key enzymes associated with AD, namely acetylcholinesterase (AChE), with inhibitory potencies in the micromolar range. Specifically, the derivatives exhibited robust inhibitory effects against AChE, with *K*_I_ values ranging from 0.63 ± 0.04 μM to 11.23 ± 1.05 μM and IC_50_ values spanning from 0.82 ± 0.06 μM to 14.75 ± 0.82 μM. Notably, all derivatives displayed superior inhibitory activity against AChE compared to the reference compound tacrine (IC_50_: 325.324 ± 28.15 μM; *K*_I_: 105.29 ± 9.67 μM). Among the series, compound 2d emerged as the most potent AR inhibitor, with a *K*_I_ value of 0.63 ± 0.04 μM, indicating its high affinity and selectivity for the enzyme. In contrast, compound 1a, while still exhibiting inhibitory activity, showed a relatively lower affinity for AChE, with a *K*_I_ value of 11.23 ± 1.05 μM. The *K*_I_ values, which reflect the binding affinity and selectivity of the inhibitors, revealed that compound 2d possessed the highest selectivity for AChE, whereas compound 1a demonstrated the lowest selectivity within the series. These findings underscore the potential of these derivatives, particularly compound 2d, as promising candidates for the development of therapeutic agents targeting AD-related enzymes. The detailed enzyme kinetic analysis, summarized in **Table 1**, provides valuable insights into the structure-activity relationships and selectivity profiles of these inhibitors, further supporting their potential for therapeutic applications.

**Table 1:** Inhibition effect on AChE of a series of imidazolium salts and tacrine as standard inhibitors.

Compound ID	AChE				
	*IC*_50_ (μM) ^a^	*R* ^2^	*K*_I_ (μM) ^a^	*R* ^2^	Inhibition type
1a	14.75 ± 0.82	0.954	11.23 ± 1.05	0.963	Uncompetitive
1b	2.21 ± 0.16	0.996	1.69 ± 0.08	0.945	Uncompetitive
1c	10.52 ± 1.08	0.961	8.05 ± 0.72	0.972	Uncompetitive
1d	1.05 ± 0.07	0.929	0.80 ± 0.06	0.948	Uncompetitive
2a	0.87 ± 0.06	0.924	0.66 ± 0.03	0.963	Uncompetitive
2b	1.02 ± 0.08	0.945	0.78 ± 0.05	0.945	Uncompetitive
2c	4.13 ± 0.25	0.934	3.16 ± 0.24	0.972	Uncompetitive
2d	0.82 ± 0.06	0.964	0.63 ± 0.04	0.948	Uncompetitive
Tacrine^b^	325.324 ± 28.15	0.975	105.29 ± 9.67	0.982	Competitive

a The test results were expressed as means of triplicate assays ± SEM.

b Tacrine was used as a positive control.

### 3.3 Antibacterial activity results

In this study, MIC values ​​lu 8 precursors including electron withdrawing quaternary ammonium group carbene precursors and NHC-M (M: Ag and Ru) complexes of these carbene precursors were synthesized and their antibacterial activities were determined. According to these results, it was found that these precursors were most effective against the gram-positive bacteria *S. aureus*. It was noteworthy that they gave the same results against the gram-negative bacteria *E. coli*, *P. aeruginosa* 1a, 1b, 2a, 2c, 2d. 1c was the most effective precursor against all the bacteria used in the study. In addition, precursor 1c gave the highest value for *P. aeruginosa*. The MIC values ​​lu the synthesized precursor compounds are shown in **Table 2**.

**Table 2:** Minimum inhibitory concentration (mg/mL) of imidazolium salts.

Compound	*S. aureus*	*E. faecalis*	*E. coli*	*P. aeruginosa*
1a	0,0250	0,05	0,2	0,2
1b	0,0125	0,05	0,2	0,2
1c	0,025	0,05	0,05	0,025
1d	0,05	0,01	0,05	0,2
2a	0,25	0,25	0,2	0,2
2b	0,25	0,05	0,1	0,2
2c	0,1	0,05	0,2	0,2
2d	0,25	0,1	0,2	0,2

### 3.4 Docking study results

In the docking study to investigate the mode of action of small molecule compounds as antimicrobial agents, we used PDB ID: 1MVT for *S. aureus* (PDB ID: 1MVT), PDB ID: 4XO8 for *E. coli* (PDB ID: 4XO8), PDB ID: 6ORI for *E. faecalis* and PDB ID: 6ORI for *P. aeruginosa*: 6ORI and for *P. aeruginosa* PDB ID: 4JVI protein structures. The ligand-protein interaction behavior was predicted using the docking score function as implemented in AutoDock 4.2.6 and the AutoDock Vina package. All calculations of the counter docking experiment for the crystal structures (PDB: 1MVT, 4XO8, 6ORI and 4JVI) are presented in **Tables 3**–**6**. All compounds were redocked into the active site without a reference inhibitor and the ligands successfully complexed with the active sites of the enzyme. The removed docked positions of the ligands were energetically minimized using a molecular mechanical force field until a gradient convergence of 0.05 kcal/mol was achieved. Poses with an initial docking score of ΔG < - 6 kcal/mol were selected. These poses were filtered according to the lowest AutoDock docking score, which was assigned to those with the lowest root mean square deviation (RMSD) relative to the reference drugs. The highest AutoDock 4.2.6 scoring function for the tested compounds was used to assess binding affinities (**Tables 3**–**6**).

**Table 3:** Binding scores and chemical bond structures in *S. aureus* bacterial structure.

Compounds	ΔG (kcal/mol)	H bond interaction	Hydrofobic interaction	Electrostatic interation
1a	-7.1	ASN464, GLU602	TYR446	MET641
1b	-5.7	SER403, SER462, ALA642, SER643	TYR446, MET641	–
1c	-7.0	HIS583, THR600	TYR446	–
1d	-7.4	ASN464, THR600, ALA642, SER643	TYR446	–
2a	-6.0	ASN464, GLN21, HIS583, SER598	TYR446	–
2b	-7.0	TYR446, ASN464	–	MET641
2c	-6.6	ASN464, THR600, ALA642, SER643	THR446, MET641	–
2d	-7.4	SER403, SER462, ASN464, THR446, SER598	HIS583	MET641

**Table 4:** Binding scores and chemical bond structures in *E. coli* bacterial structure.

Compounds	ΔG (kcal/mol)	H bond interaction	Hydrofobic interaction	Electrostatic interation
1a	-6.3	VAL94, ASN96, PRO102	LYS76, PRO104	–
1b	-5.2	SER78, PRO104	LYS101	–
1c	-7.4	SER39, SER97, LYS101	LYS76, PRO102, PRO104, ALA106	–
1d	-7.1	SER78, VAL94	PRO102, TRP103, PRO104	LYS101
2a	-6.2	ASP37, LYS101	PRO102, PRO104	–
2b	-6.9	SER39, SER97, LYS101	LYS76, PRO104, ALA106	–
2c	-6.2	VAL94, SER97, LYS101	LYS101, PRO104	–
2d	-6.7	SER97	VAL93, LYS101, PRO102, PRO104	LYS101

**Table 5:** Binding scores and chemical bond structures in *E. faecalis* bacterial structure.

Compounds	ΔG (kcal/mol)	H bond interaction	Hydrofobic interaction	Electrostatic interation
1a	-7.3	ASP421	ILE423	–
1b	-6.0	GLN120, ASN159	ILE160, MET213	–
1c	-7.2	ASP421	ILE423	–
1d	-7.7	GLY230, GLU232	TYR72, PRO229, LYS292, ALA342, VAL388	ASP290
2a	-6.2	LYS200	ALA204	–
2b	-7.2	THR239, ASP240, GLN241	PHE199, LYS200, ALA204	–
2c	-6.8	ASP421	ILE423	–
2d	-7.4	LYS200, ALA204	LEU207	–

**Table 6:** Binding scores and chemical bond structures in *P. aeruginosa* bacterial structure.

Compounds	ΔG (kcal/mol)	H bond interaction	Hydrofobic interaction	Electrostatic interation
1a	-6.8	–	LEU208, ILE236	–
1b	-5.3	LEU197	LEU208,ILE236	–
1c	-7.1	–	VAL170, LEU208,ILE236, TYR258, ILE263	–
1d	-7.4	LEU207	ALA168, VAL170, LEU208, VAL211, ILE236, TYR258	–
2a	-6.1	GLN194, LEU207, LEU208	ILE236	–
2b	-7.2	–	VAL170, LEU189, LEU208, VAL211, ILE236, TYR258	–
2c	-6.4	LEU197	LEU208, VAL211, ILE236	–
2d	-7.0	SER255	ALA168, VAL170, LEU207, ILE236, TYR258	–

The amino acid residues of the bacterial structures associated with compounds 1a-2d are shown in **Figures 5**–**8**. The targeting of 1a-2d to the active pocket regions of the bacterial structures is shown in **Figures 9**–**12**.

**Figure 5:**
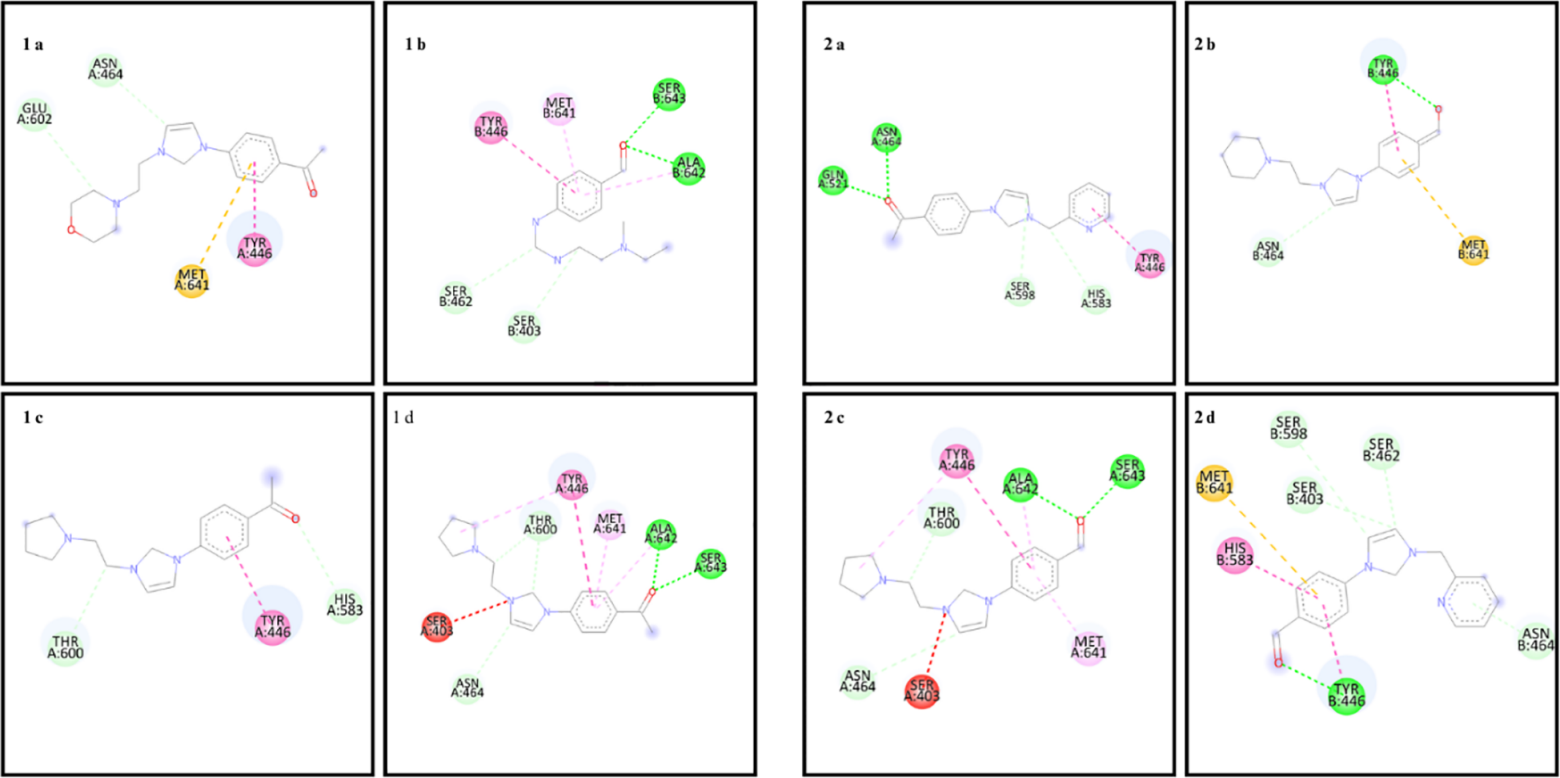
Chemical binding targets of 1a-2d in *S. aureus* bacterial structure.

**Figure 6:**
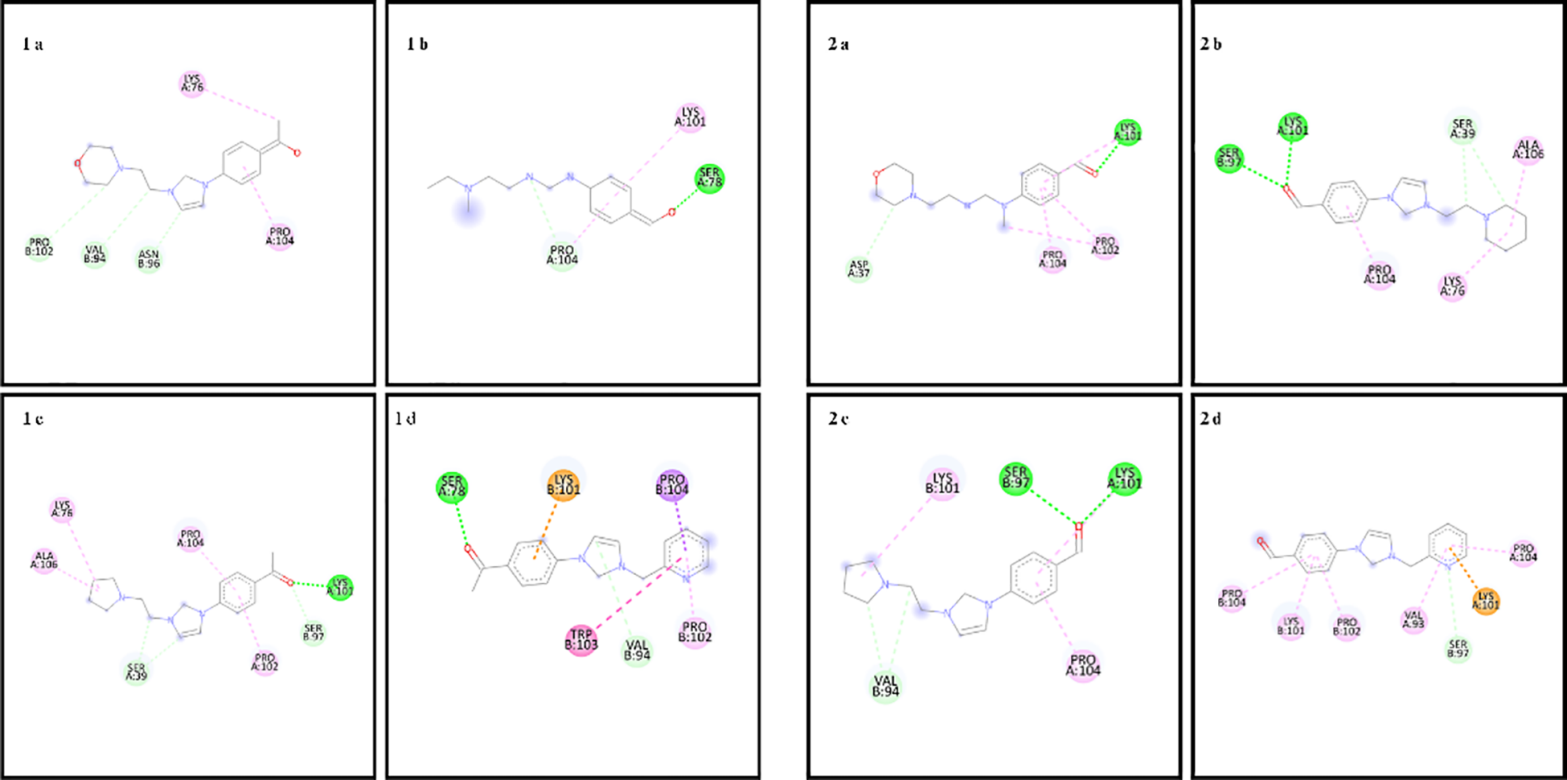
Chemical binding targets of 1a-2d in *E. coli* bacterial structure.

**Figure 7:**
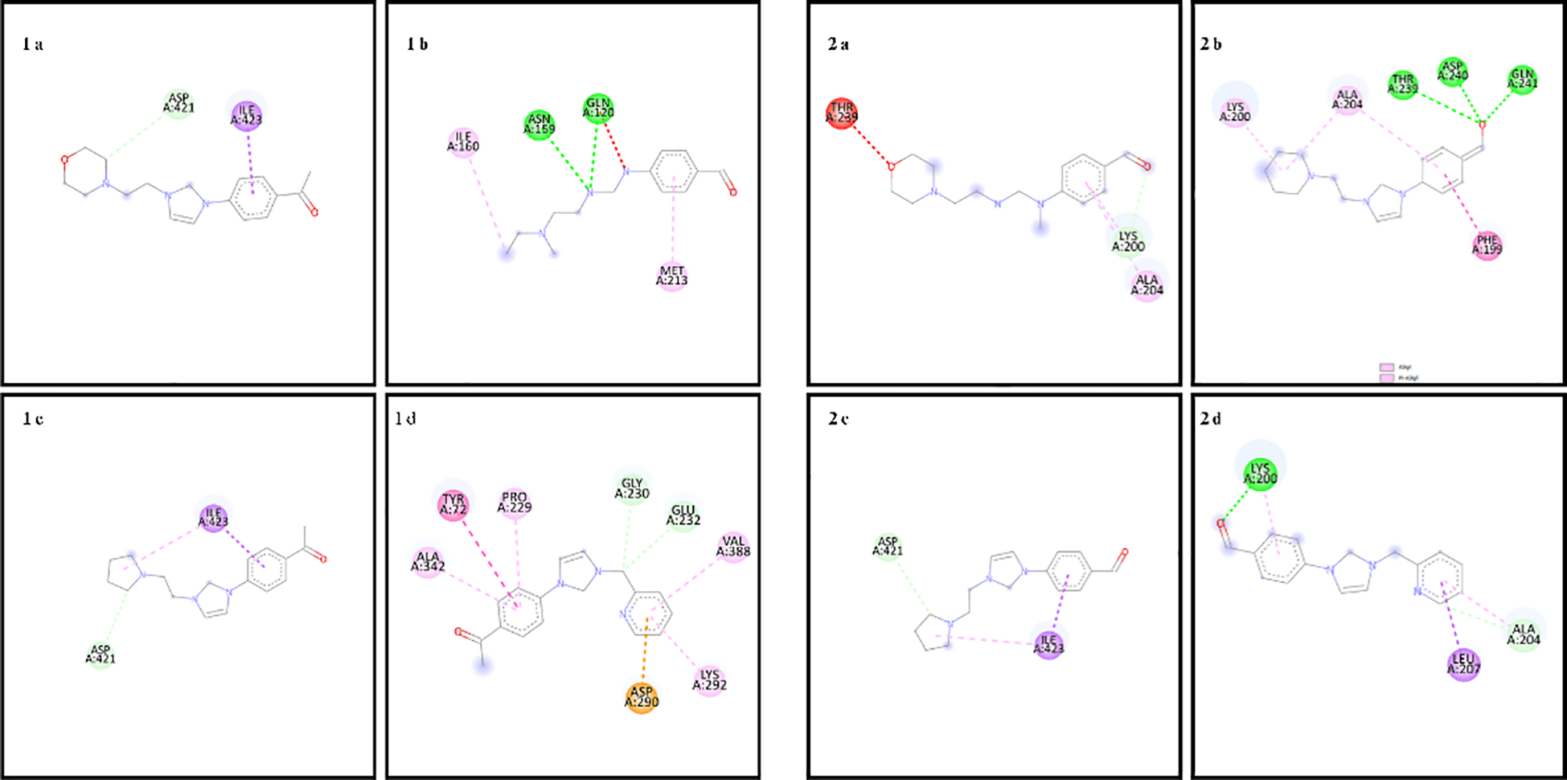
Chemical binding targets of 1a-2d in *E. Faecalis* bacterial structure.

**Figure 8:**
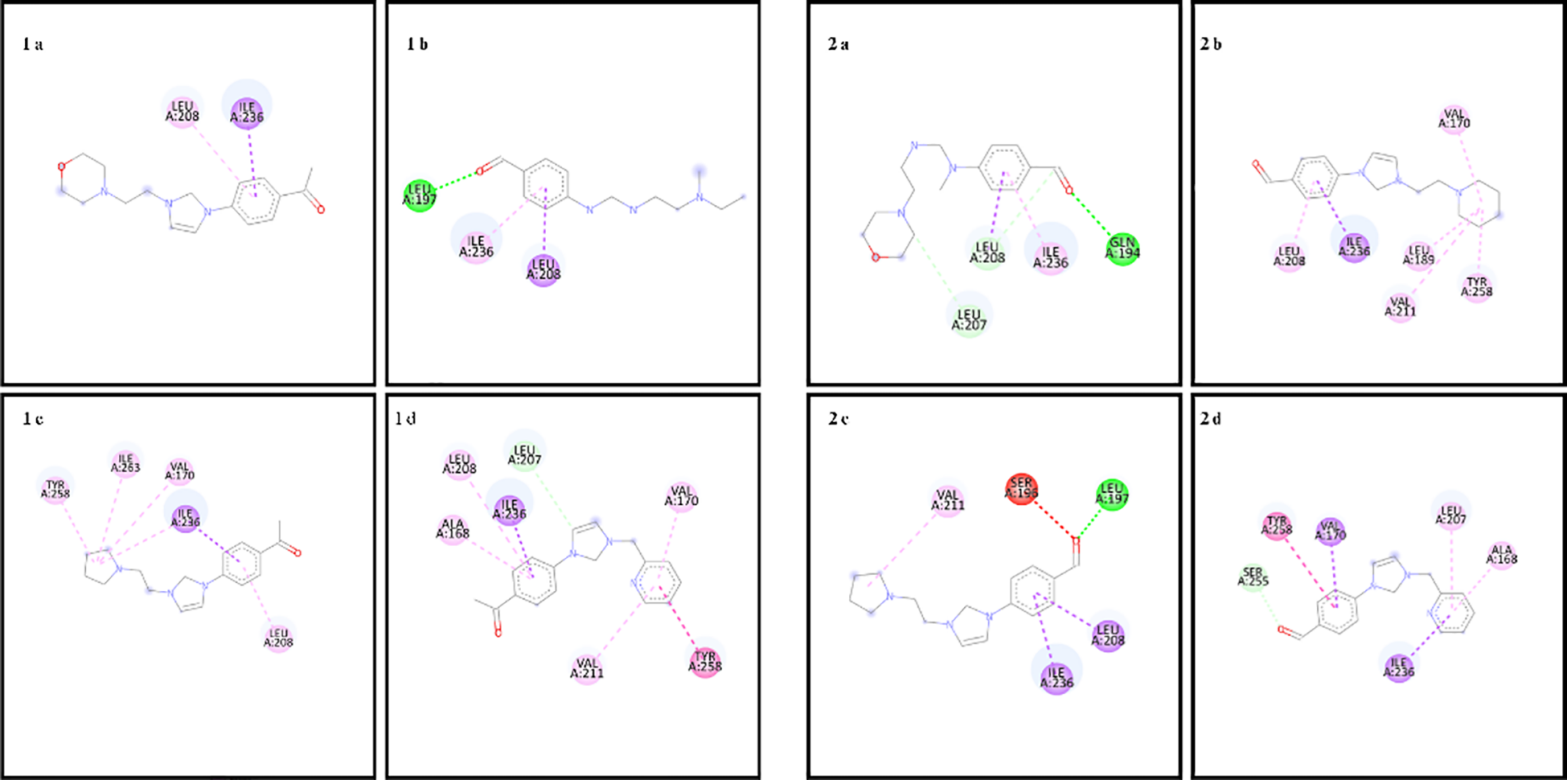
Chemical binding targets of 1a-2d in *P. aeruginosa* bacterial structure.

**Figure 9:**
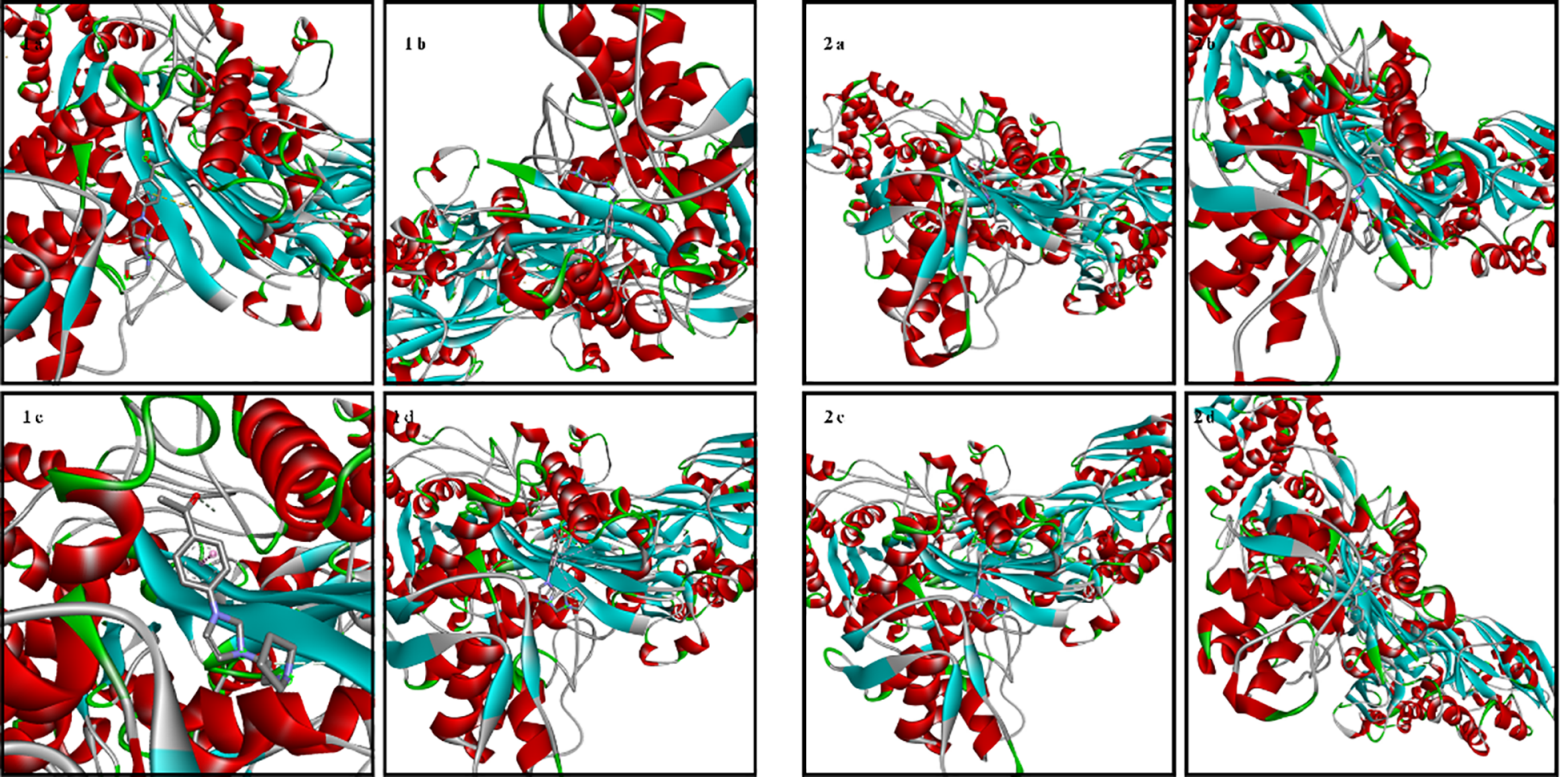
Binding model of 1a-2d in the bacterial structure of *S. aureus*.

**Figure 10:**
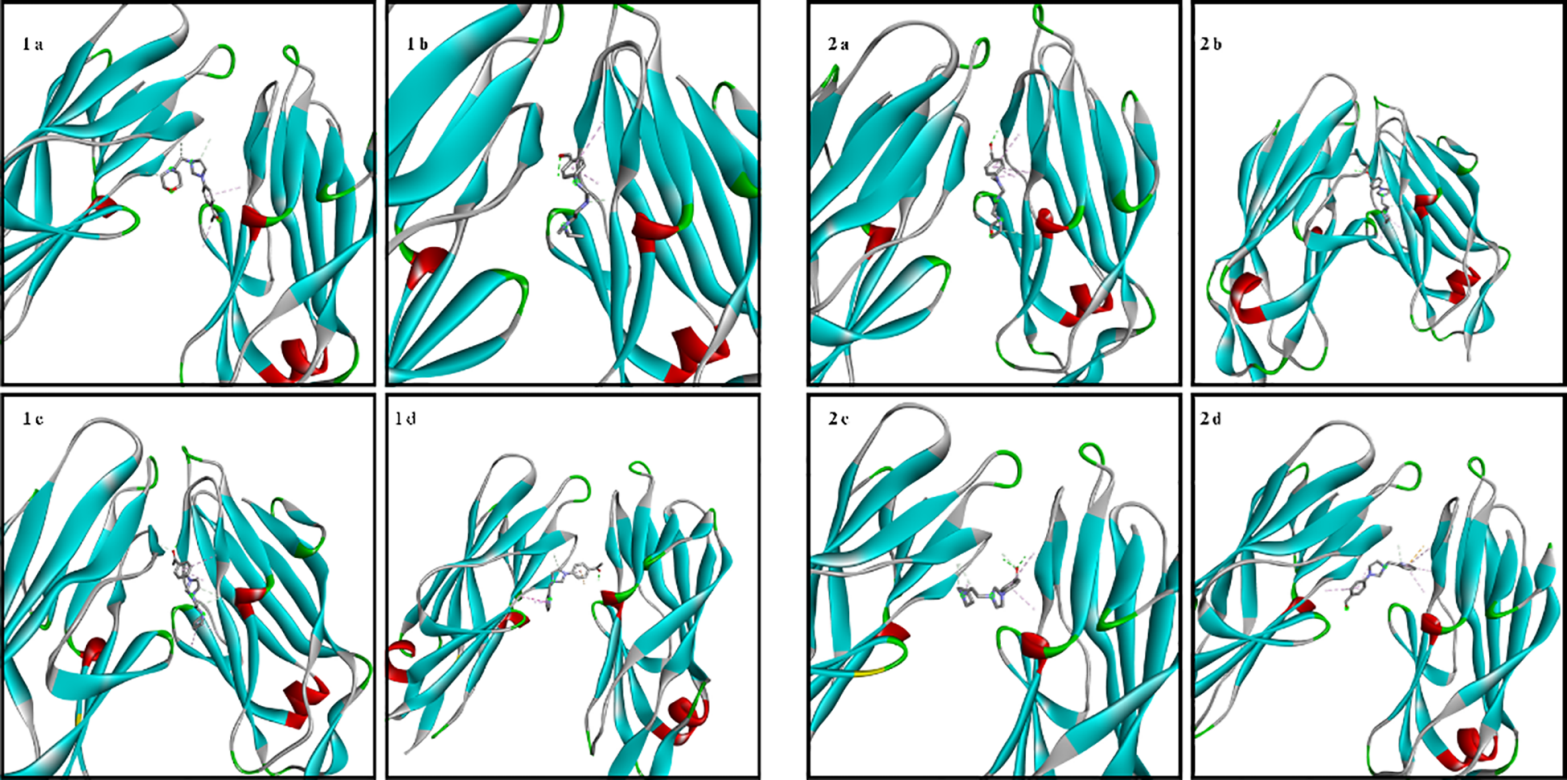
Binding model of 1a-2d in the bacterial structure of *E. Coli*.

**Figure 11:**
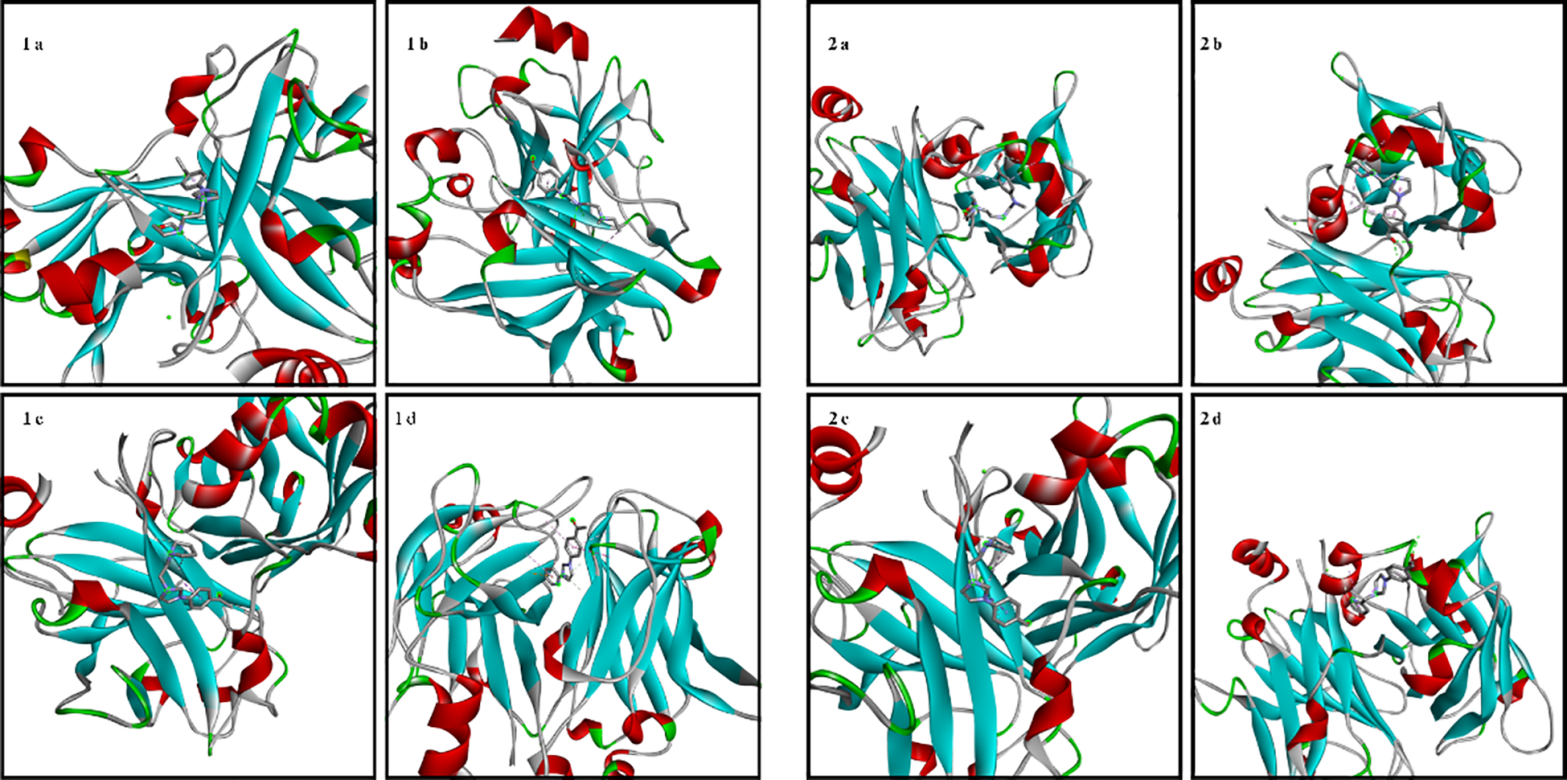
Binding model of 1a-2d in the bacterial structure of *E. Faecalis*.

**Figure 12:**
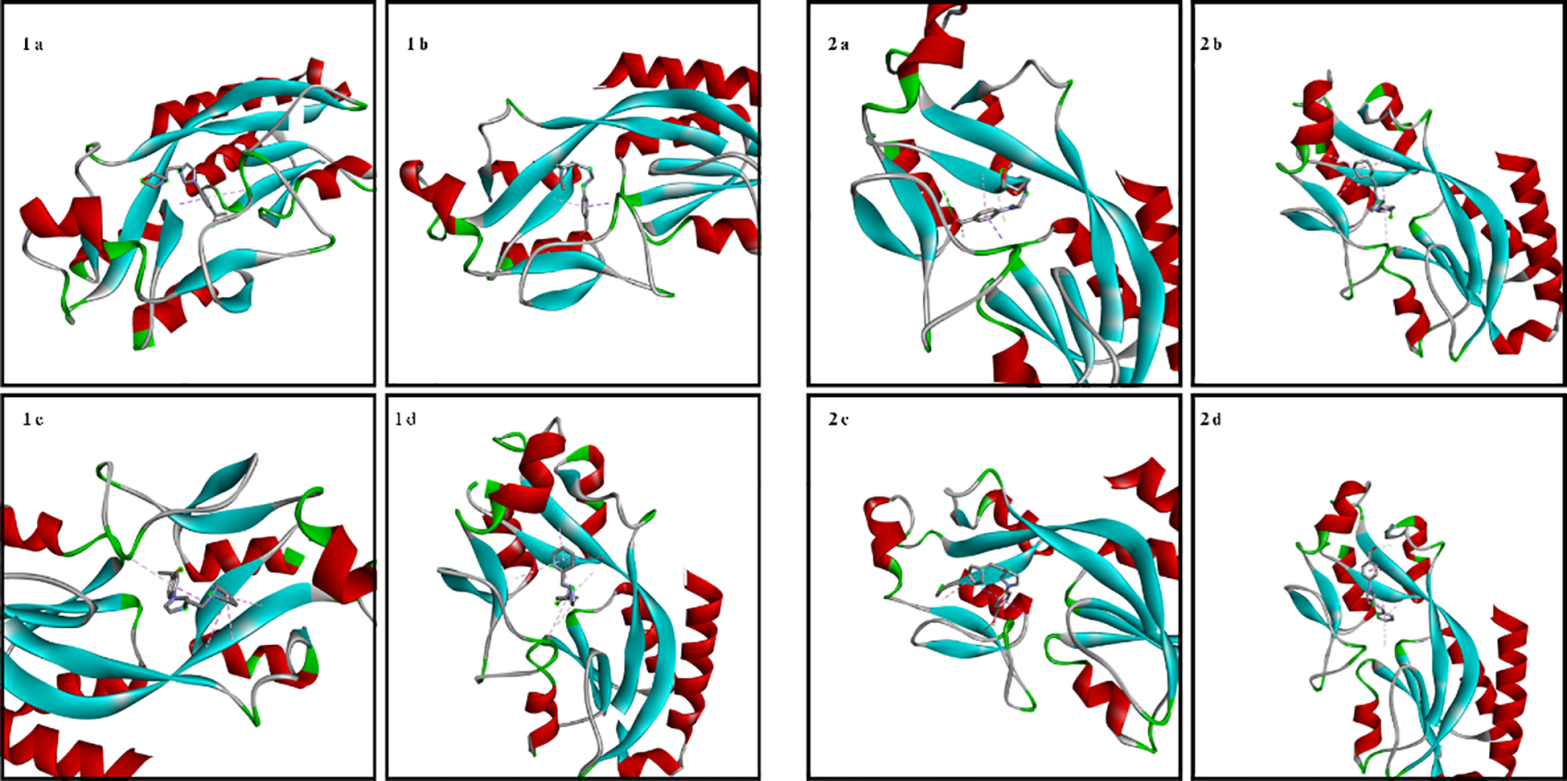
Binding model of 1a-2d in the bacterial structure of *P. aeruginosa*.

## 4 Discussion

In this study, it was hypothesized that imidazolium salts carrying two positive charges could serve as suitable alternatives to quaternary ammonium compounds, which are widely used as antiseptics, disinfectants, and antimicrobial agents, due to their tunable hydrophobic, hydrophilic, and electrostatic properties. Therefore, our study involves the synthesis and investigation of the biological properties of two series of imidazolium salts. These compounds incorporate bioactive heterocyclic rings such as piperidine, morpholine, pyrrole, and pyridine, introduced by alkylation at the third nitrogen atom of the 1-(4-acetyl/formylbenzyl)imidazole ring. In this study, eight imidazolium salts were synthesized, all of which are novel. The imidazolium salts were synthesized in high yields ranging from 66% to 87%. The structures of all synthesized salts were characterized by using ^1^H NMR, ^13^C NMR, FTIR spectroscopic methods, and elemental analysis techniques. These salts, which are stable against air and moisture, are very soluble in polar organic solvents such as ethyl alcohol, dimethylformamide and dimethylsulfoxide, but are insoluble in nonpolar solvents such as hexane, toluene and diethyl ether. When the ^1^H NMR spectra of these 1a-d salts are examined, the characteristic 2-CH proton signals of imidazolium salts containing the acetyl group (1a-d) are seen at 8.20, 8.81, 8.49 and 10.90 ppm, respectively, proving the formation of these salts. The protons belonging to the aromatic group (-C_6_H_4_COCH_3_) in compounds 1a-d give doublet peaks at approximately 7.49 to 8.23 ppm. The hydrogens of the imidazole ring were observed as siglets between 7.25 and 8.52 ppm. Benzylic CH_2_ protons in compound 1d give siglet peaks of 5.77 ppm.

The characteristic 2-CH proton signals of imidazolium salts (2a-d) containing the formyl group were also seen at 10.02, 10.13, 10.12 and 10.10 ppm, respectively. The formation of the imidazolium salts were established by the appearance of the characteristic proton peak belonging to the 2-CH group in the imidazole ring. Protons belonging to the aromatic group (-C_6_H_4_COCHO) to which the formyl group in compounds 2a-d is attached give doublet peaks at around 7.99 to 8.21 ppm. The proton of the formyl group was observed at around 10 ppm. Benzylic CH_2_ protons in compound 2d give siglet peaks of 5.75 ppm.

In the ^13^C NMR spectra of all imidazolium salts (1a-d and 2a-d) bearing 4-acetylphenyl and 4-formylphenyl groups, the characteristic 2-C peaks of imidazolium salts were seen at 142.7, 139.9, 142.4, 139.3, 141.9, 139.4, 139.4 and 139.5 ppm, respectively. Benzylic CH_2_ carbon in compounds 1d and 2d give siglet peaks of 54.5 and 56.5 ppm, respectively. The carbon of the formyl group in compounds 2a-d is observed at approximately 192.7 ppm, while the carbonyl carbon in compounds 1a-d is observed at approximately 197.4 ppm.

4-Acetylphenyl, 4-formylphenyl and a second heterocyclic substituent containing imidazolium FTIR spectra of the salts were taken. The FTIR data for imidazolium salts of the 4-formylphenyl group revealed a characteristic ν(C–N) band at 1603, 1605, 1557, and 1501 cm^−1^, while ν(C=O) band was observed at 1672, 1682, and 1636 cm^-1^ for 1a–d, respectively. At the same time, the FTIR data for imidazolium salts bearing the 4-acetylphenyl group show that a characteristic ν(C–N) band is observed at 1603, 1604, and 1602 cm^−1^ for 1a, 1c and 1d, while ν(C=O) band was observed at 1700, 1696 cm^-1^ for 2b and 2d, respectively. All spectroscopic data obtained appear to be compatible with the literature (Sarı et al., 2018; Erdemir et al., 2018; Türker et al., 2018; Behçet et al., 2018; Yiğit et al., 2018; Bal et al., 2021).

The compounds synthesized in this study exhibited remarkable AChE inhibitory activity at the micromolar level, demonstrating significantly enhanced potency compared to the results reported in previous studies. For instance, in a prior investigation, 4-aminobenzenesulfonamide derivatives were synthesized and evaluated for their AChE inhibitory effects, with *K*_I_ constants ranging from 2.54 ± 0.22 µM to 299.60 ± 8.73 µM (Işık et al., 2019). Similarly, another study focused on the synthesis of novel pyridine 2,4,6-tricarbohydrazide derivatives, which displayed AChE inhibitory activity with an IC_50_ value of 50.2 ± 0.8 µM (Riaz et al., 2015). In contrast, the compounds developed in the current study achieved micromolar-range IC_50_ values, highlighting their superior inhibitory efficacy.

The results suggest that these compounds act as uncompetitive inhibitors of AChE, highlighting their potential as promising candidates for the development of therapeutic agents targeting neurodegenerative diseases such as Alzheimer’s, where AChE inhibition is a critical strategy to mitigate disease progression.

In this study, MIC values ​​were obtained from eight carbene precursors (NHC-M) carrying quaternary ammonium groups with electron-withdrawing properties depending on their antibacterial activities. Literature studies have shown that imidazolium salts have the expected weak MIC values ​​ (Messali et al., 2013; Riduan and Zhang, 2013; Coleman et al., 2012). According to these results, these prodrugs were the most effective against the gram-positive bacterium *S. aureus*. This can be explained by the presence of the protective membrane on the outer surface of Gram-positive bacteria. On the other hand, it was observed that the synthesized compounds 1a, 1b, 2a, 2c, and 2d were quite active against Gram-negative bacteria (*E. coli, P. aeruginosa*). It was noteworthy that they gave the same results against precursors 1a, 1b, 2a, 2c, and 2d. In the study, compound 1c was the most effective precursor against all the bacteria used in the study. This situation is related to the length of the alkyl chain of the imidazolium salts. Also, the highest value for *P. aeruginosa* was also given by precursor 1c. Our research results are consistent with the literature (Demberelnyamba et al., 2004).

The docking results in the *S. aureus* bacterial structure showed that the binding affinities of compounds 1a-2d were 1d>2d>1a>1a>1c=2b>2c>2a>1b, respectively. *E. coli*, 1c>1d>2b>2d>1a>2a=2c>1b, in *E. faecalis* the order was 1d>2d>1a>1c=2b>2c>2a>1b and finally in *P. aeruginosa* the order was 1d>2b>1c>2d>1a>2c>2a>1b. When the bacterial results were compared with the molecular docking results, it was found that compounds 1c, 1a, 1b, 2a, 2c and 2d showed good binding affinity to bacteria according to the docking results. Results supporting the bacterial study were observed.

## 5 Conclusion

Imidazolium salts containing groups with electron withdrawing properties on the first nitrogen atom (4-acetylphenyl and 4-formylphenyl) and heterocyclic substituents with bioactive properties (morpholine, pyrrolidine, pyrrole and pyridine) on the second nitrogen atom were synthesized by quaternization method. The synthesized salts were characterized by various physical and analytical techniques such as elemental analysis, melting point, ^1^H and ^13^C NMR and FTIR. The presence of two positive charges in the synthesized compounds will allow these compounds to interact electrostatically with enzymes and bacteria. In addition, these salts have extensive hydrogen bonding networks and wing-tip N-functionalizations, which impart special properties such as hydrophilicity on azolium salts.

In this study, the inhibitory potentials of compounds synthesized on metabolically important cholinergic enzymes (AChE) in the formation of AD were analyzed and demonstrated. As a result, it was determined that compounds (1a-d and 2a-d) showed inhibitory properties especially on Acetylcholinesterase enzyme (AChE) related to AD. This substantial improvement in potency underscores the potential of the newly synthesized compounds as highly effective AChE inhibitors, positioning them as promising candidates for further development in the treatment of neurodegenerative disorders. Finally, it is important to investigate and know the active metabolic enzyme inhibitory effects of such compounds found in naturally sourced algae rather than synthetic inhibitors used for treatment purposes. In this study, eight imidazalium precursors were synthesized and their inhibitory effects on Gram positive and negative bacteria were examined. It was evaluated that the synthesized compounds had an inhibitory effect especially on Gram negative bacteria. The obtained results show that antimicrobial activity should be taken into consideration in the design of imidazalium salts and their evaluation in clinical and industrial applications.

## Data Availability

The datasets presented in this study can be found in online repositories. The names of the repository/repositories and accession number(s) can be found in the article/**Supplementary Material**.
