# Supplementary material for: Imidazolium salts carrying two positive charges: design, synthesis, characterization, molecular docking, antibacterial and enzyme inhibitory activities
Source: Front Cell Infect Microbiol. 2025 Jul 18;15:1579916. doi: 10.3389/fcimb.2025.1579916 (PMC12313567; doi:10.3389/fcimb.2025.1579916)
Supplement: Supplementary file 1 [file Table1.docx]

**SUPPORTING INFORMATION**

**^1^H NMR and ^13^C NMR spectra of compound 1a**


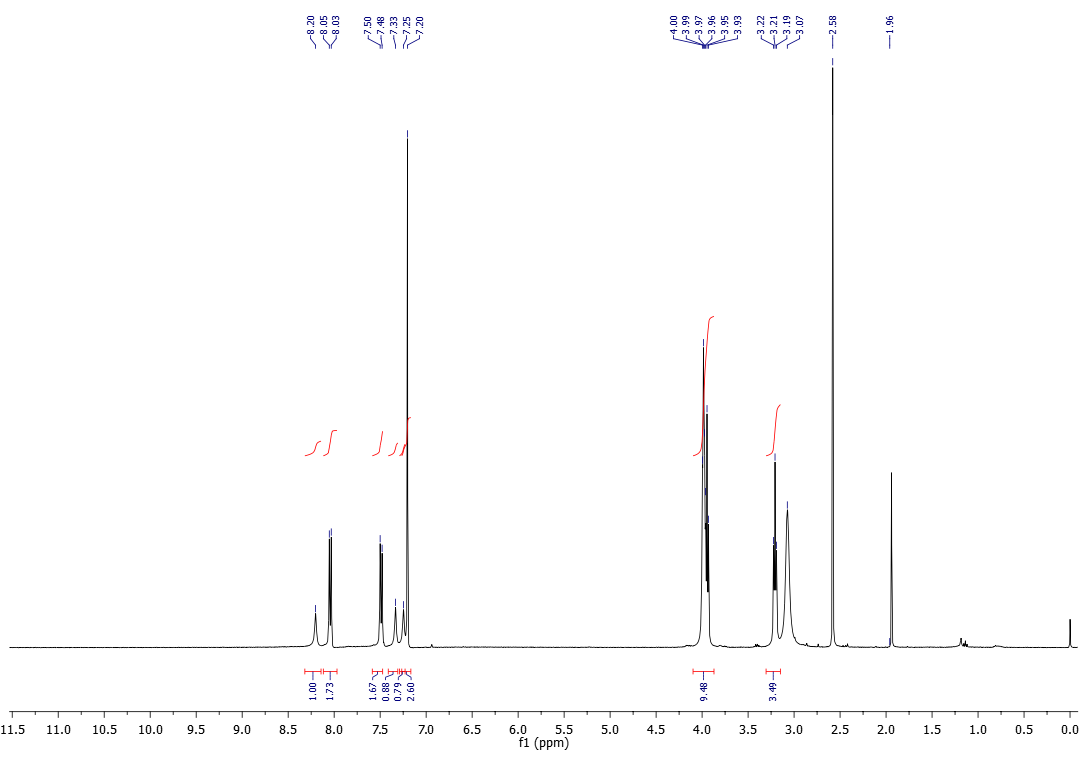


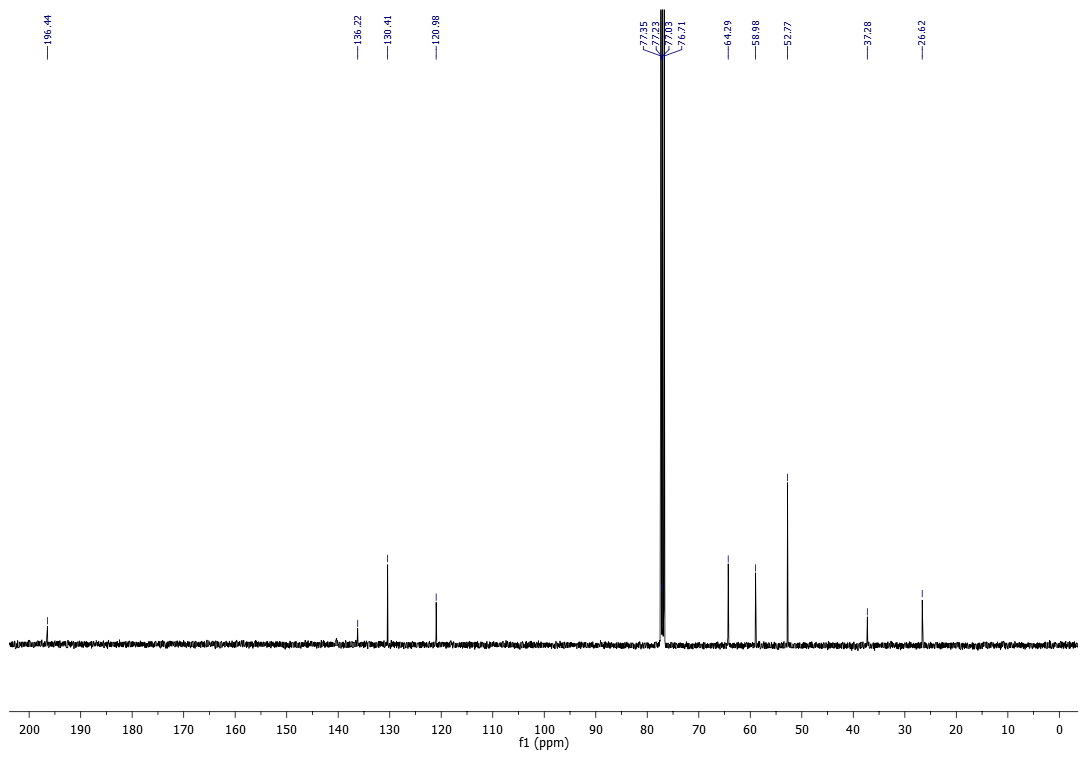


**^1^H NMR and ^13^C NMR spectra of compound 1b**


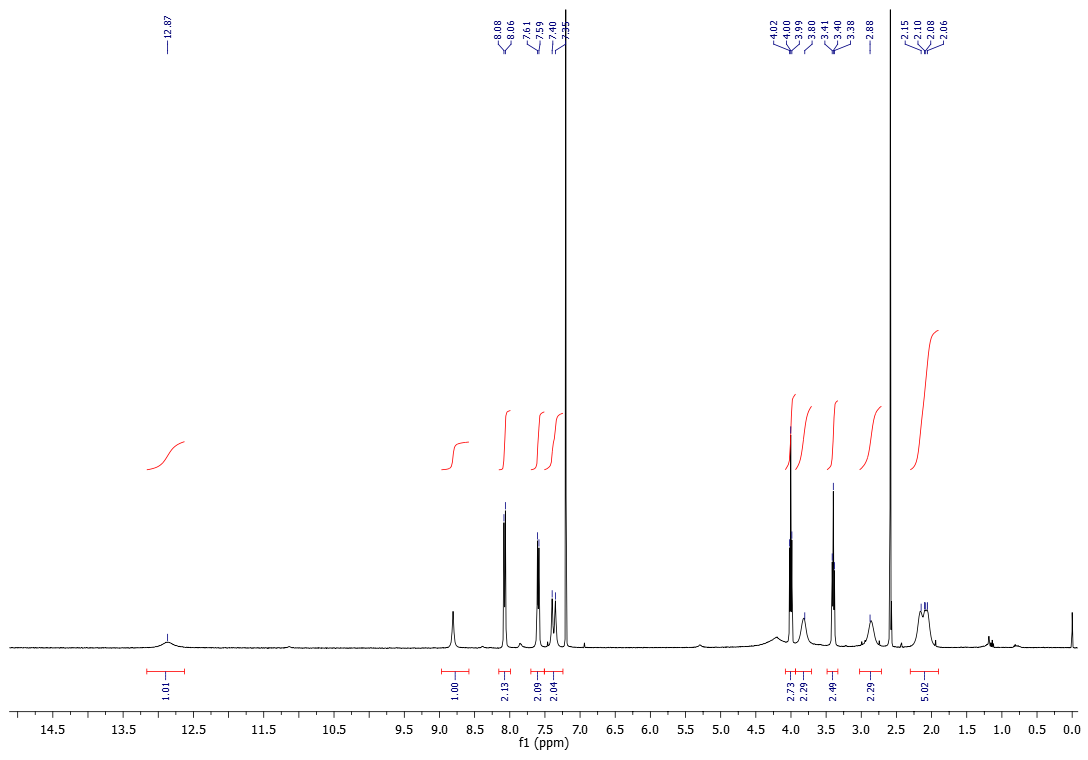


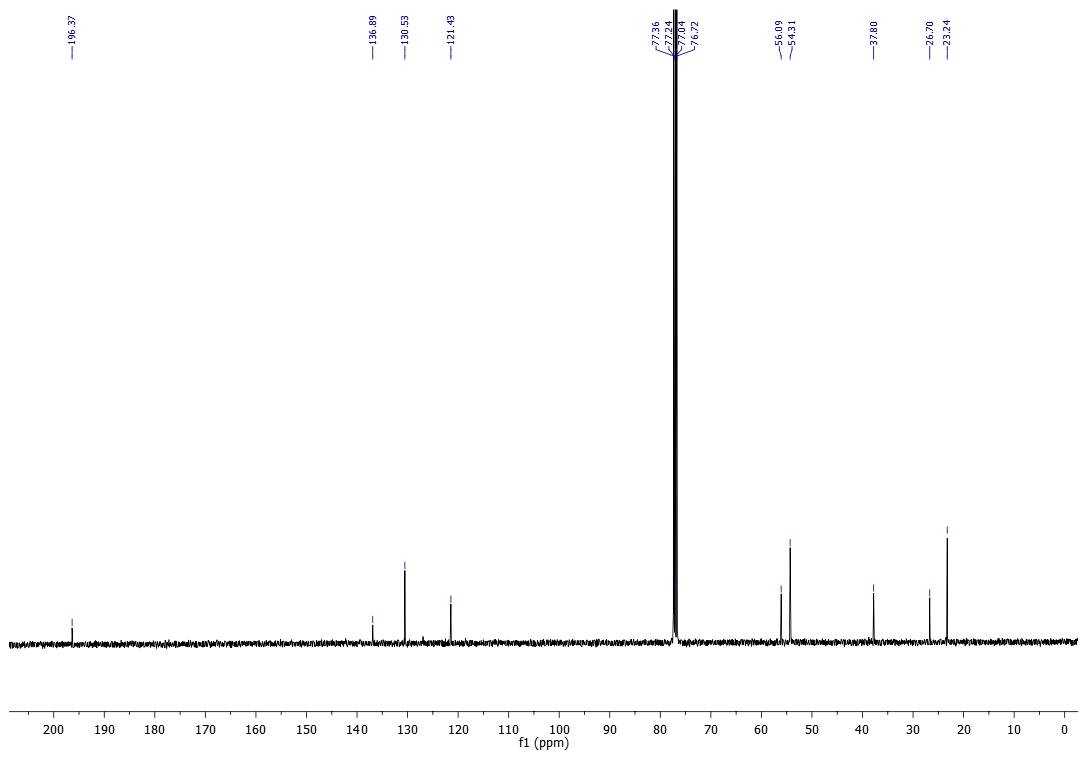


**^1^H NMR and ^13^C NMR spectra of compound 1c**


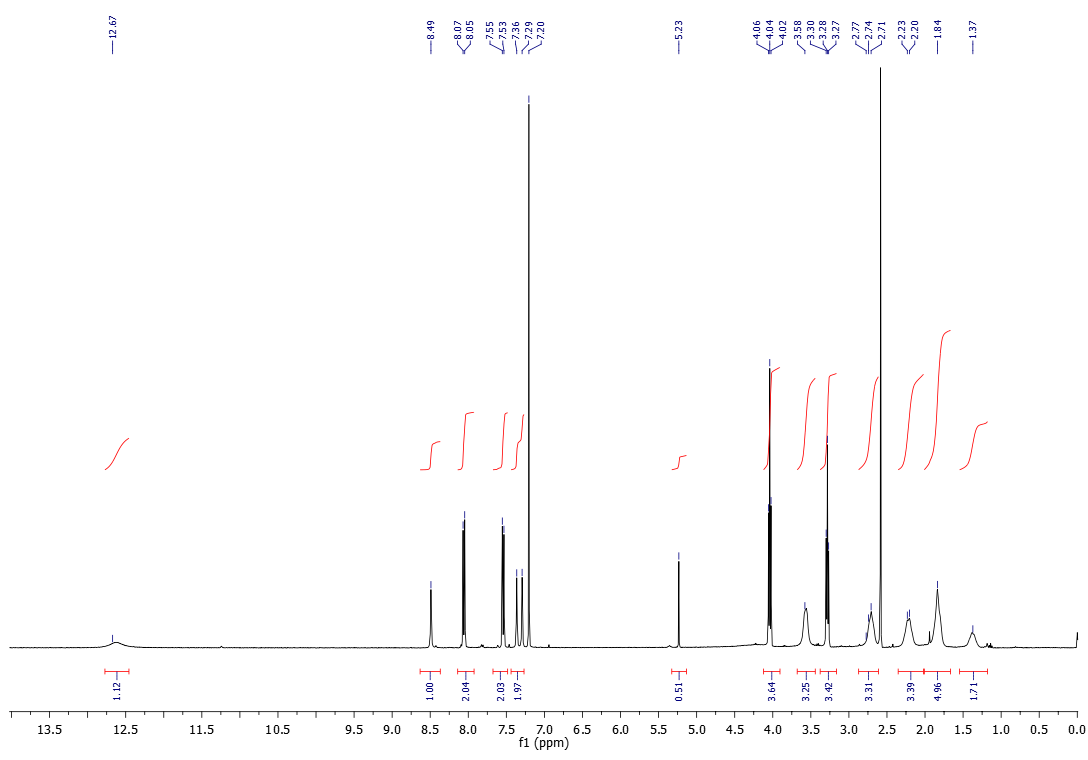


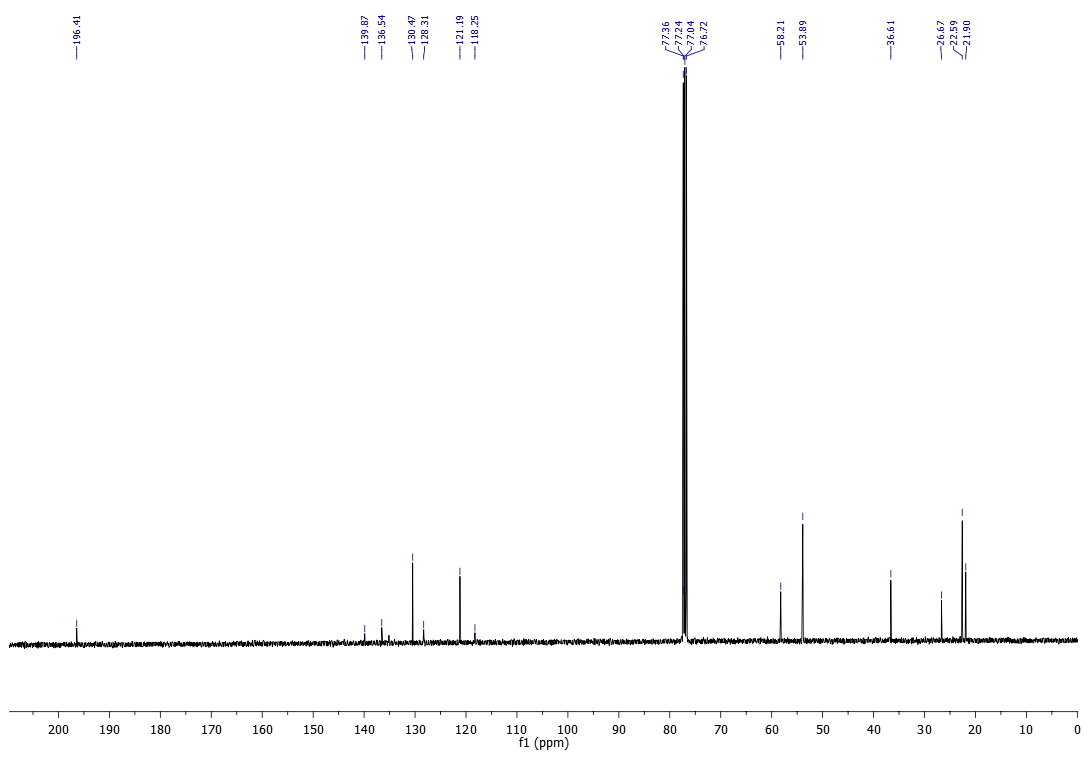


**^1^H NMR and ^13^C NMR spectra of compound 1d**


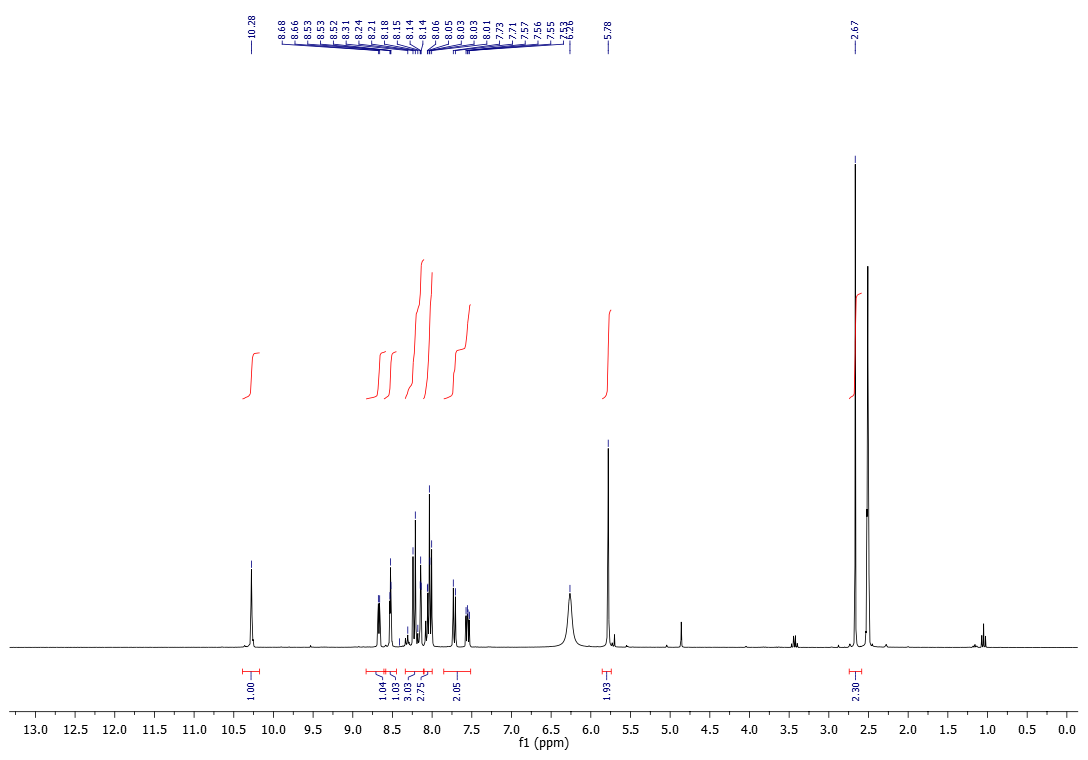


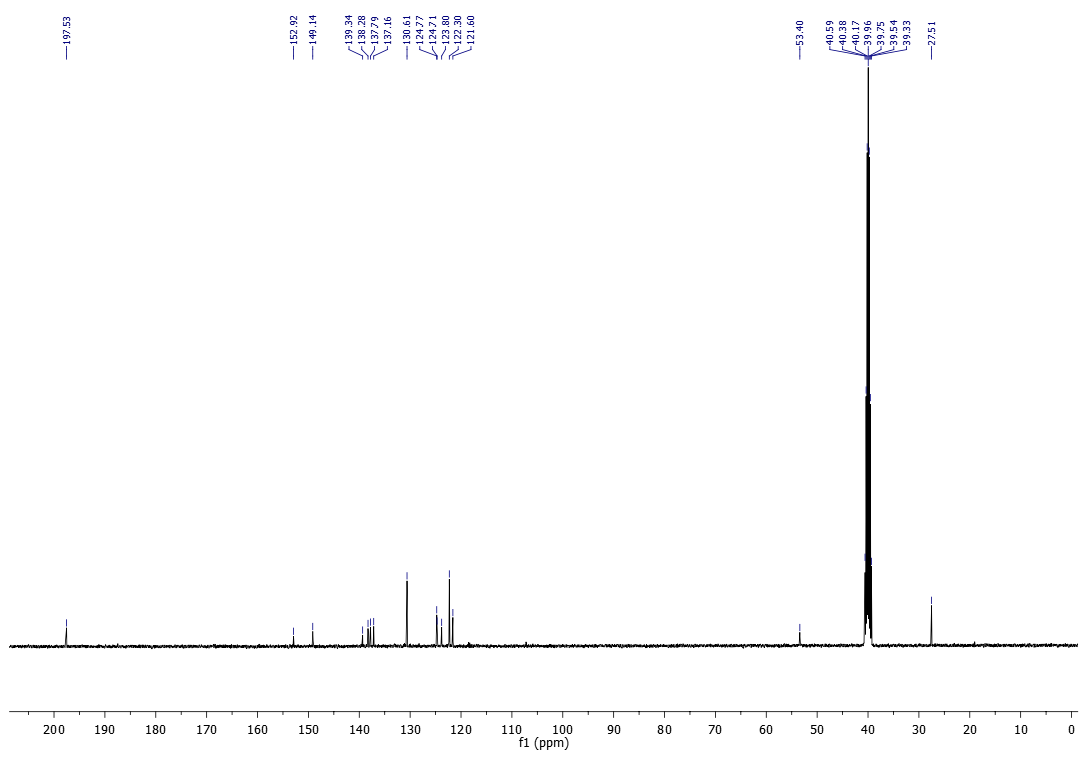


**^1^H NMR and ^13^C NMR spectra of compound 2a**


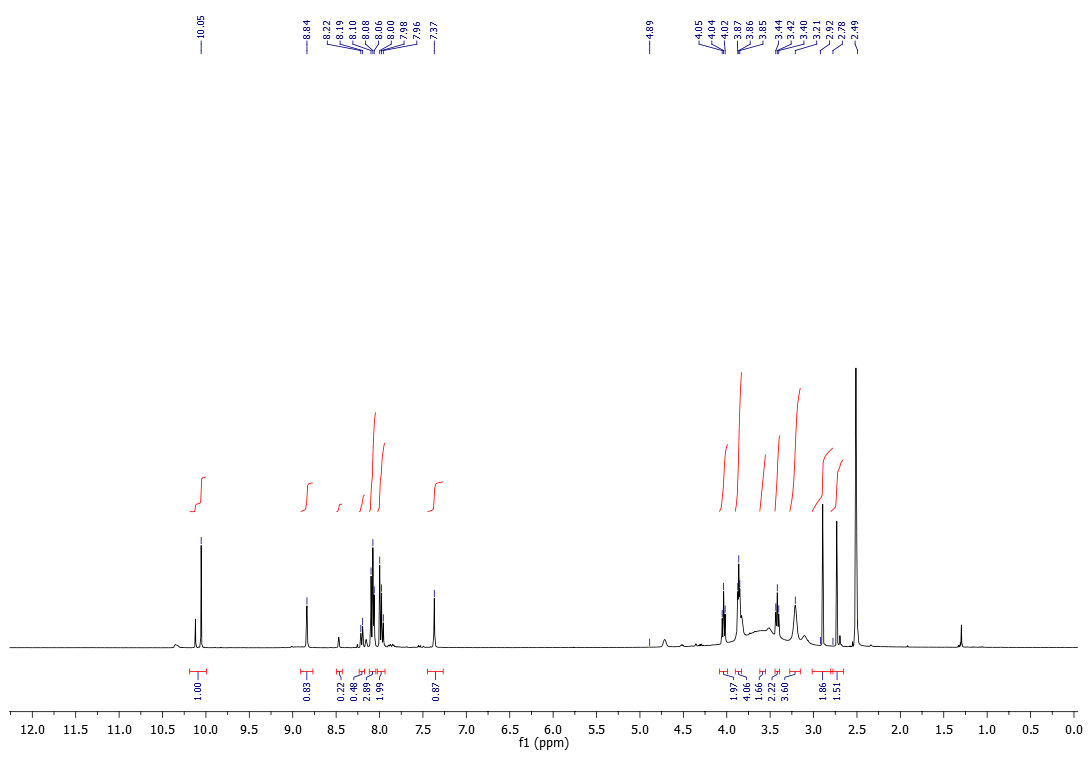


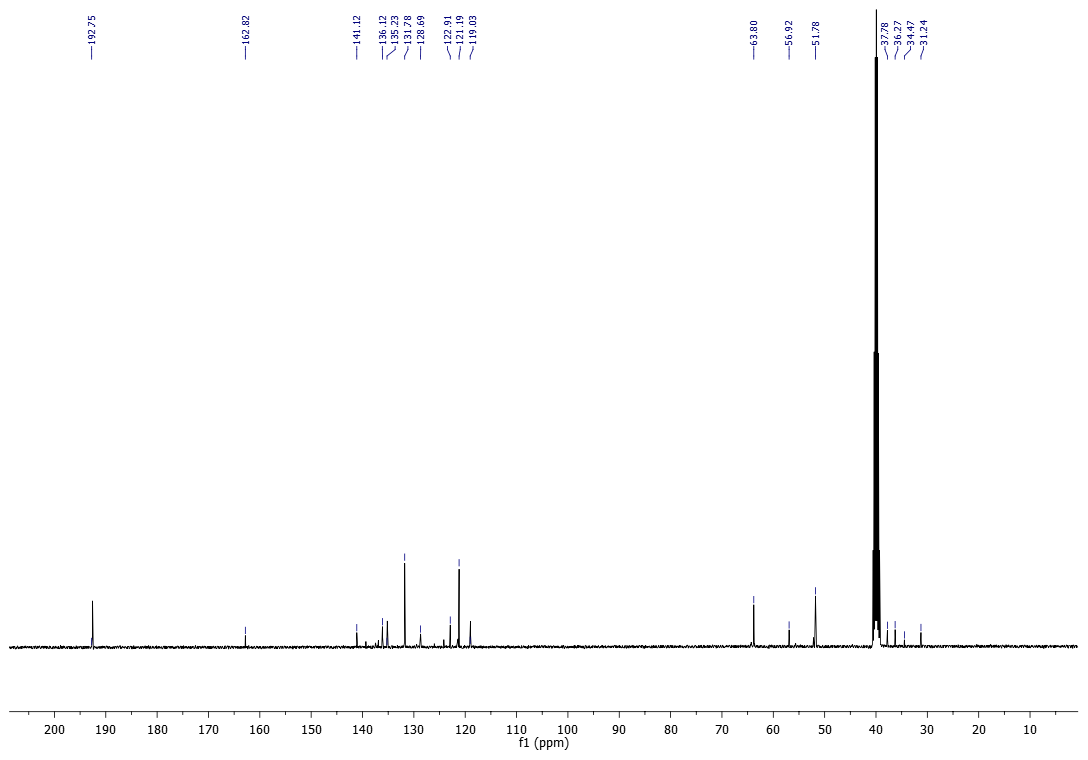


**^1^H NMR and ^13^C NMR spectra of compound 2b**


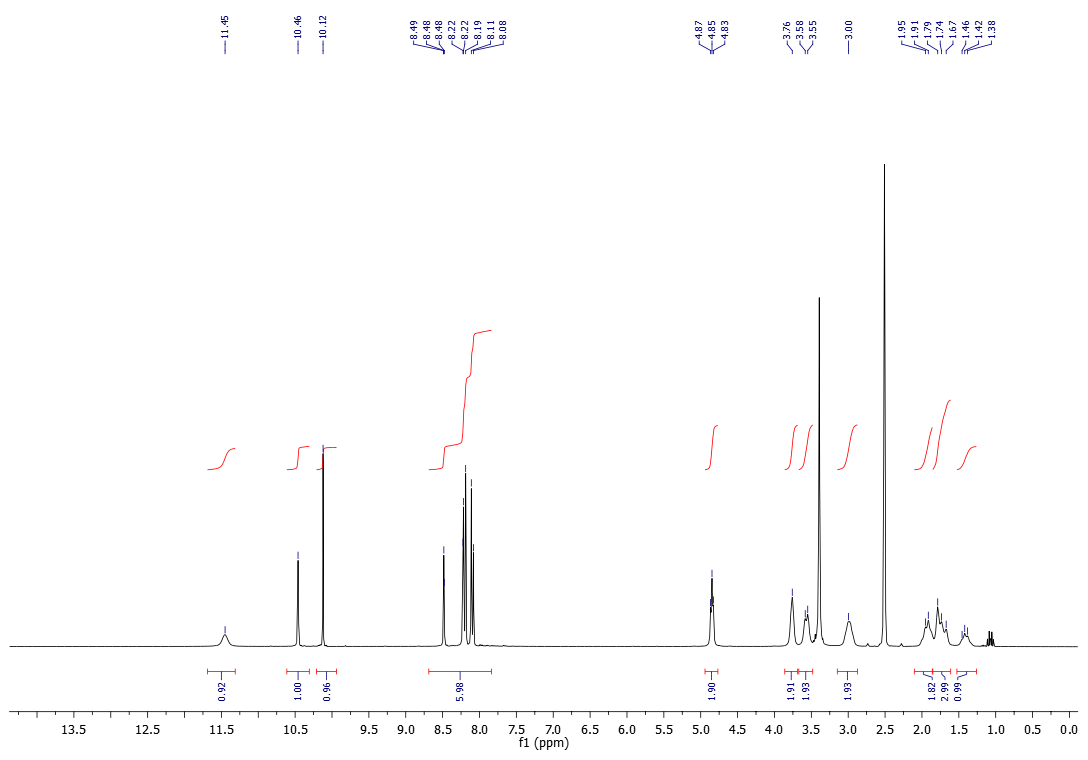


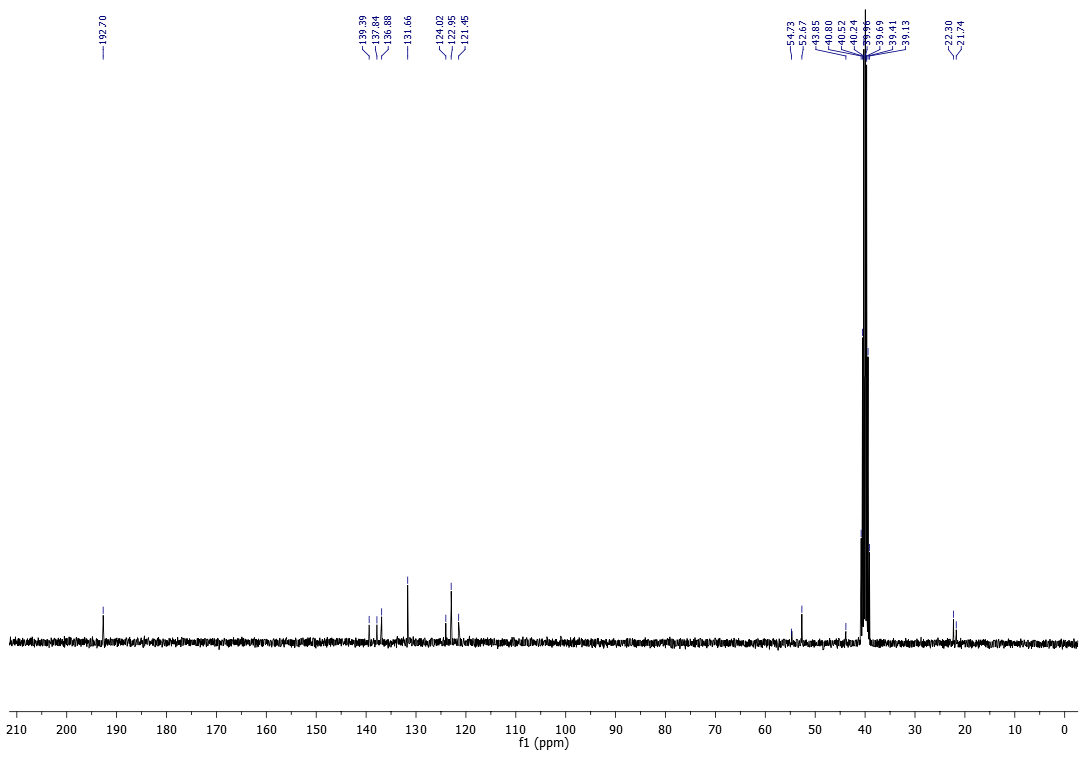


**^1^H NMR and ^13^C NMR spectra of compound 2c**


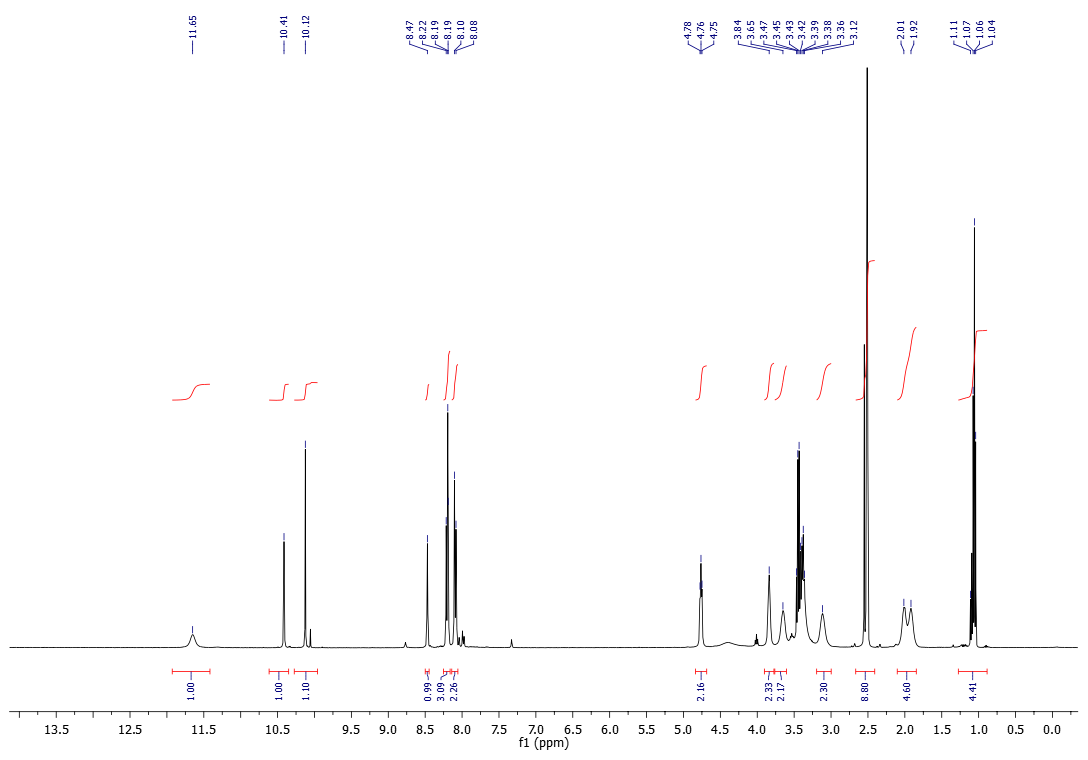


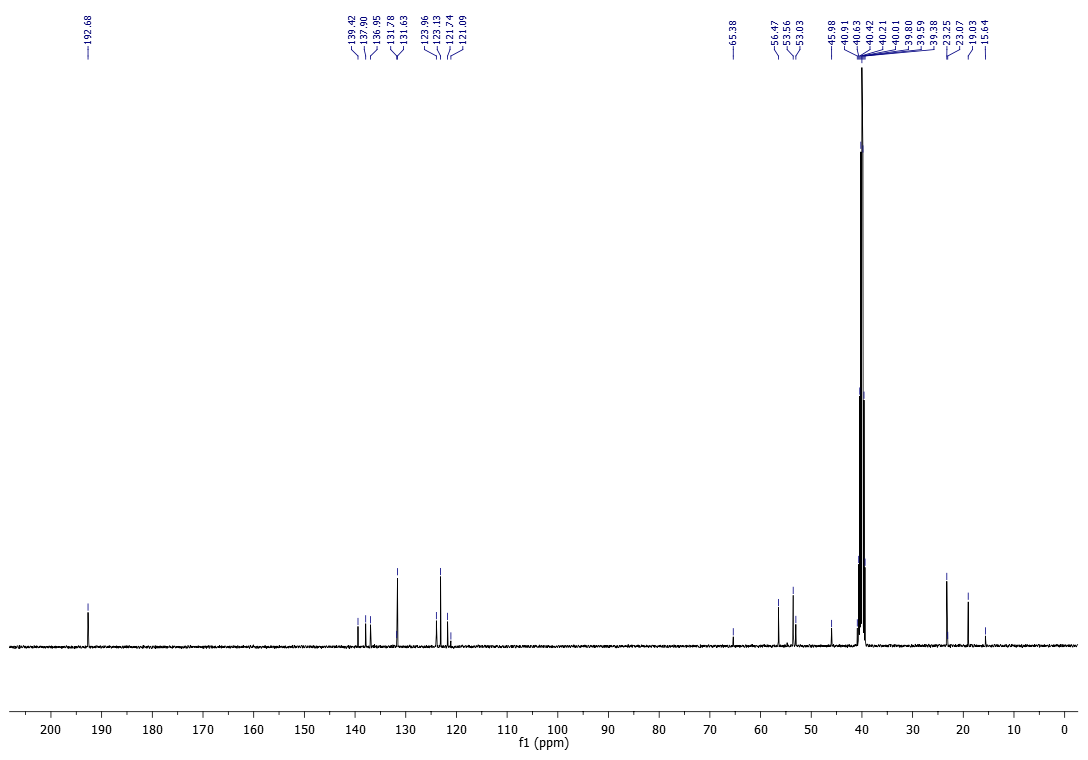


**^1^H NMR and ^13^C NMR spectra of compound 2d**


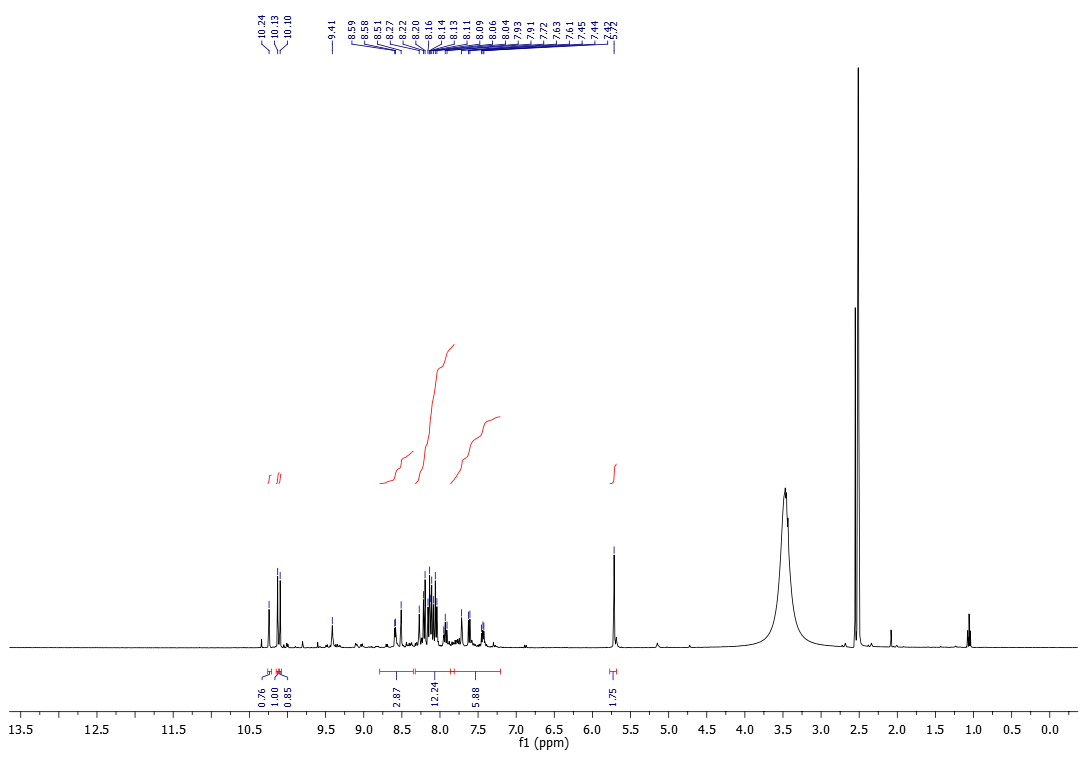


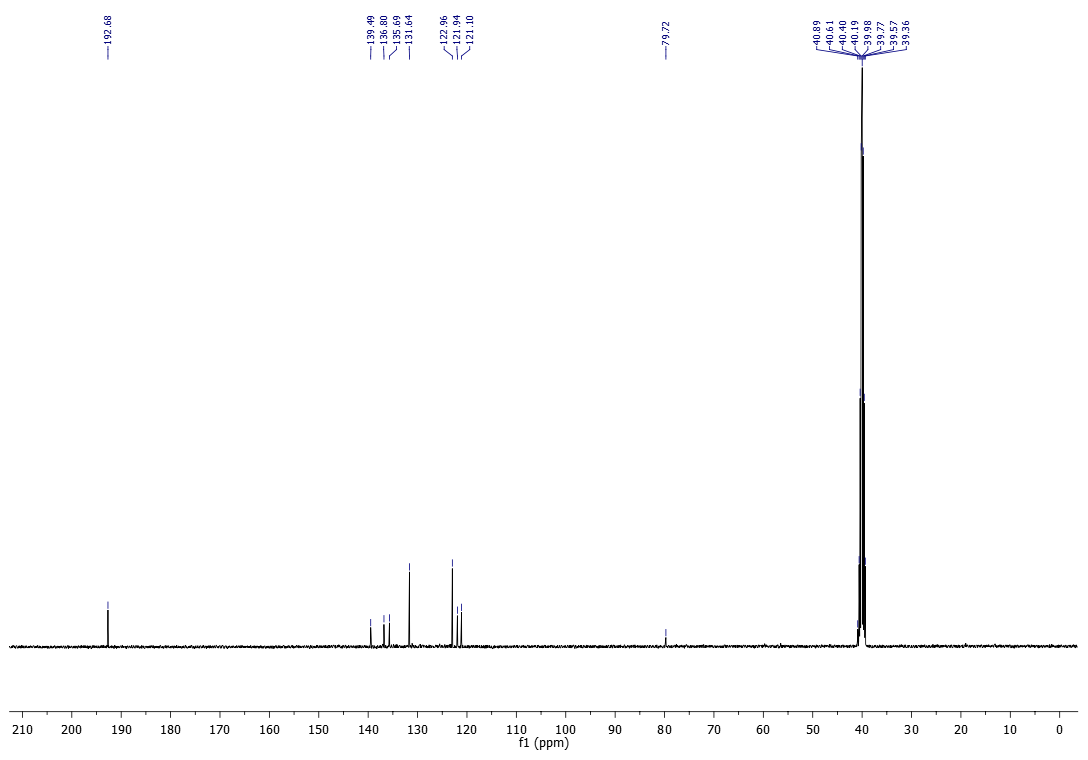


**FTIR spectrum data for compound 1a**

**FTIR spectrum data for compound 1c**

**FTIR spectrum data for compound 1d**

**FTIR spectrum data for compound 2b**

**FTIR spectrum data for compound 2d**
